# Prognostic Value of mRNAsi/Corrected mRNAsi Calculated by the One-Class Logistic Regression Machine-Learning Algorithm in Glioblastoma Within Multiple Datasets

**DOI:** 10.3389/fmolb.2021.777921

**Published:** 2021-12-06

**Authors:** Mingwei Zhang, Hong Chen, Bo Liang, Xuezhen Wang, Ning Gu, Fangqin Xue, Qiuyuan Yue, Qiuyu Zhang, Jinsheng Hong

**Affiliations:** ^1^ Department of Radiotherapy, Cancer Center, The First Affiliated Hospital of Fujian Medical University, Fuzhou, China; ^2^ Institute of Immunotherapy, Fujian Medical University, Fuzhou, China; ^3^ Key Laboratory of Radiation Biology of Fujian Higher Education Institutions, The First Affiliated Hospital, Fujian Medical University, Fuzhou, China; ^4^ Department of Gastrointestinal Surgery, Fujian Provincial Hospital, Fuzhou, China; ^5^ Nanjing University of Chinese Medicine, Nanjing, China; ^6^ Nanjing Hospital of Chinese Medicine Affiliated to Nanjing University of Chinese Medicine, Nanjing, China; ^7^ Department of Radiology, Fujian Cancer Hospital & Fujian Medical University Cancer Hospital, Fuzhou, China

**Keywords:** glioblastoma, mRNAsi, OCLR, prognosis, stemness indices

## Abstract

Glioblastoma (GBM) is the most common glial tumour and has extremely poor prognosis. GBM stem-like cells drive tumorigenesis and progression. However, a systematic assessment of stemness indices and their association with immunological properties in GBM is lacking. We collected 874 GBM samples from four GBM cohorts (TCGA, CGGA, GSE4412, and GSE13041) and calculated the mRNA expression-based stemness indices (mRNAsi) and corrected mRNAsi (c_mRNAsi, mRNAsi/tumour purity) with OCLR algorithm. Then, mRNAsi/c_mRNAsi were used to quantify the stemness traits that correlated significantly with prognosis. Additionally, confounding variables were identified. We used discrimination, calibration, and model improvement capability to evaluate the established models. Finally, the *CIBERSORTx* algorithm and ssGSEA were implemented for functional analysis. Patients with high mRNAsi/c_mRNAsi GBM showed better prognosis among the four GBM cohorts. After identifying the confounding variables, c_mRNAsi still maintained its prognostic value. Model evaluation showed that the c_mRNAsi-based model performed well. Patients with high c_mRNAsi exhibited significant immune suppression. Moreover, c_mRNAsi correlated negatively with infiltrating levels of immune-related cells. In addition, ssGSEA revealed that immune-related pathways were generally activated in patients with high c_mRNAsi. We comprehensively evaluated GBM stemness indices based on large cohorts and established a c_mRNAsi-based classifier for prognosis prediction.

## 1 Introduction

Glioblastoma multiforme (GBM), is the most common and most malignant glial tumour (Young et al., 2015). There is no clear way to prevent GBM; the disease can be very difficult to treat, and a cure is often not possible. The typical treatment, which involves surgery, chemotherapy, and radiation therapy, (Gallego, 2015), may slow cancer progression and reduce signs and symptoms. However, cancer usually recurs despite treatment (Gallego, 2015). The most common length of survival following diagnosis is 12–15 months, with less than 3–5% of the patients surviving longer than 5 years (Gallego, 2015). Without treatment, the survival time is typically 3 months (McNeill, 2016). Therefore, developing and applying signatures or biomarkers that can effectively predict the prognosis of these patients is of vital importance. A good initial Karnofsky Performance Score (KPS), the methylation of the O6-methylguanine-DNA methyltransferase (*MGMT*) promoter, and mutations in isocitrate dehydrogenase 1 (*IDH1*) are associated with longer survival (Krex et al., 2007; Martinez et al., 2007; Burgenske et al., 2019; Chaddad et al., 2019). The above signatures or biomarkers can be used either alone or in combination to predict the prognosis of GBM (Molenaar et al., 2014). However, their predictive capacity is rather low and a new index is needed.

Stem-like cells, which are characterised by the self-renewal properties and therapeutic resistance, play crucial roles in various cancers, (Kaushal and Ramakrishna, 2020), especially in GBM (Wang et al., 2018). Although cancer stem-like cells are very important for prognosis in GBM, (Turaga et al., 2020), there are still some shortcomings and complications in quantifying these cells. The stemness features have been extensively studied using artificial intelligence and deep learning methods. (Pan et al., 2019). A good example is the calculation of the mRNA expression-based stemness index (mRNAsi) with the one-class logistic regression (OCLR) machine-learning algorithm (Sokolov et al., 2016; Malta et al., 2018). Tathiane M. Malta *et al.* used mRNAsi for the first time to reflect the degree of oncogenic dedifferentiation (Malta et al., 2018). They also found tumour heterogeneity at the single-cell level by measuring the mRNAsi and concluded that a lower mRNAsi correlated with better prognosis in various cancers. (Malta et al., 2018). The prognostic value of the mRNAsi differs among different cancers. Moreover, we have previously shown that the prediction performance of a single mRNAsi-based signature is not good in primary lower-grade glioma, (Zhang et al., 2020b), partly because the tissue biopsy samples are often mixed with non-tumour tissues (bulk tissues). This means that the expression data on which the mRNAsi is based may be contaminated with non-tumour information. Thus, tumour purity may solve this issue (Xia et al., 2020).

It remains unclear whether the mRNAsi is an independent prognostic indicator in GBM and whether the predictive capacity of mRNAsi is better than that of existing factors such as the mutational status of *IDH1* and the methylation status of *MGMT*. Previous studies have shown that the combination of clinical features with signatures or biomarkers can significantly improve prognosis prediction, (Zhang et al., 2020b; Zhang et al., 2020c), but this has not been verified with the mRNAsi, let alone the corrected mRNAsi (c_mRNAsi), which is acquired using ‘Estimation of STromal and Immune cells in MAlignant Tumours using Expression data’ (ESTIMATE) (Yoshihara et al., 2013) to calculate tumour purity. Whether c_mRNAsi can predict GBM better than mRNAsi is unknown. Furthermore, although Tathiane M. Malta *et al.*(Malta et al., 2018) analysed cancer stemness quite extensively, this was done in almost 12,000 samples of 33 tumour types from only The Cancer Genome Atlas (TCGA) (Hoadley et al., 2014). Thus, overfitting was inevitable and the generalisation ability of the mRNAsi was not evaluated. Therefore, the prognostic value of the mRNAsi in GBM needs to be validated in other independent databases, such as the Chinese Glioma Genome Atlas (CGGA) and Gene Expression Omnibus (GEO) (Barrett et al., 2013).

In this study, we used mRNA expression data and the OCLR machine-learning algorithm to simultaneously examine the independent prognostic value of mRNAsi/c_mRNAsi in TCGA, CGGA, and two GEO datasets. We compared mRNAsi/c_mRNAsi directly and evaluated the model improvement ability. Then, we applied the latest *CIBERSORTx* tool (Newman et al., 2019) to evaluate the relationship between mRNAsi/c_mRNAsi and immune cell infiltration and conducted single sample gene set enrichment analysis (ssGSEA) to comprehensively examine its prognostic value and relationship with the immune microenvironment.

## 2 Materials and Methods

### 2.1 Data Acquisition

RNA-sequencing data (level 3) of 158 patients with GBM from TCGA and 279 patients with GBM from the CGGA were obtained. The data from TCGA were downloaded from the University of California Santa Cruz (UCSC) Xena website (https://xena.ucsc.edu/). Transcript abundances were measured in fragments per kilobase of transcript per million mapped reads (FPKM). We only included patients who had adequate clinical and pathological data. Then, to uncover the practicability and accuracy of independent prognostic factors for GBM, samples from the TCGA and CGGA cohorts were applied as training and validation cohorts, respectively. Moreover, we included two GEO datasets (GSE4412 (Freije et al., 2004) and GSE13041 (Lee et al., 2008)) with more than 100 samples and follow-up data as our external validation data. The characteristics of the patients from the databases are presented as means ± standard deviations (continuous variables that satisfied the normal distribution), median, minimum, maximum and quartile (continuous variables that did not satisfy the normal distribution), and percentage (categorical variables), as appropriate.

### 2.2 mRNAsi/c_mRNAsi Acquisition

The mRNAsi was calculated using the OCLR machine-learning algorithm (Malta et al., 2018). Tumour purity was evaluated with ESTIMATE (Yoshihara et al., 2013) and c_mRNAsi was obtained by dividing the mRNAsi by tumour purity (Zhang et al., 2020b). The gene expression-based mRNAsi/c_mRNAsi was represented using β values ranging from zero (no gene expression) to one (complete gene expression).

### 2.3 Analysis of Independent Prognostic Factors

#### 2.3.1 The Relationship Between mRNAsi/c_mRNAsi and Overall Survival (OS)

To explore the effect of mRNAsi/c_mRNAsi on OS of patients with GBM, we used locally weighted scatterplot smoothing (Lowess) algorithm to flexibly evaluate the association of mRNAsi/c_mRNAsi with OS. The results were obtained as a fitting smooth curve. When the curve was linear, mRNAsi/c_mRNAsi was included as a continuous variable; otherwise, mRNAsi/c_mRNAsi was included as a dichotomous variable in the subsequent analysis.

#### 2.3.2 Survival Analysis

When the variables were analysed as dichotomous variables, the optimal cut-off for each index with the associated hazard of OS was identified by log-rank statistics in a survfit model, using the *cutp* function of the *survMisc* package. Then, patients with GBM were included into either the high or the low group according to the optimal cut-off. Next, Kaplan-Meier analysis with log-rank test was conducted to estimate the survival curves of each group and to compare the prognosis between different groups, by using the *survival* package.

#### 2.3.3 Identification of Confounding Variables

Residual confounding variables refer to incomplete adjustment for factors related to both exposure and outcome (Kernan et al., 2000). The confounding variables that may influence the OS of patients with GBM need to be identified. To estimate the magnitude of the effect of mRNAsi/c_mRNAsi on GBM, we used the Cox proportional hazards model. The regression coefficient changed more than 10% when the adjustment variables were included or not included or when those with *p* < 0.1 in the univariate analysis with OS were considered as confounding variables to be adjusted (adjusted I/II model) (Kernan et al., 2000). The common covariates in TCGA were age, gender, IDH, radiotherapy, chemotherapy, and subtype. In addition, 1p19q and MGMTp were common covariates in CGGA but without subtype. Afterward, an interaction test and a stratified analysis (Soria et al., 2015) of the association between mRNAsi/c_mRNAsi and OS were conducted in both the non-adjusted model and adjusted I model (identified confounders). A two-tailed *p* < 0.05 was considered statistically significant. Empower (www.empowerstats.com; X&Y solutions Inc., Boston, MA) and R (http://www.R-project.org) were used for the abovementioned statistical analyses.

### 2.4 Construction and Comparison of Prognostic Models

#### 2.4.1 Model Establishment

After confirming the effect of mRNAsi/c_mRNAsi on OS, we further evaluated and compared the benefit of five different models, including the prognostic model constructed by well-established clinical factors (model 1), model 1 integrated with mRNAsi (model 2)/c_mRNAsi (model 3), and single mRNAsi (model 4)/c_mRNAsi (model 5).

#### 2.4.2 Model Evaluation and Nomogram

We used discrimination, calibration, and model improvement capability to assess the performance of the different models. Discrimination was evaluated through the receiver operating characteristic (ROC) curve, (Zhou et al., 2019), concordance index (C-index) (Harrell et al., 1996) and the prediction error and decision curve analysis (DCA) curves (Kerr et al., 2016). Notably, the enhanced bootstrap method with 500 resamples was used for internal validation (Wang et al., 2019). Discrimination and calibration were evaluated by apparent and adjusted C-index and Brier Score. Finally, model improvement capability was evaluated by applying net reclassification improvement (NRI) and integrated discrimination improvement (IDI) using the *survIDINRI* package (Pencina et al., 2008). After the best model was identified, the *regplot* package was employed to construct the nomogram.

#### 2.4.3 External Validation

We applied the data from CGGA and GEO as external validation. In CGGA, as described above, we performed mRNAsi/c_mRNAsi acquisition, independent prognostic factors analysis, and prognostic model construction and comparison. It should be noted that because the clinical information in TCGA and CGGA was not identical, common covariates were not the same. GSE4412 (Freije et al., 2004) and GSE13041, (Lee et al., 2008), which constitute GEO, were also applied for the external effect validation of mRNAsi/c_mRNAsi on OS. Similarly, patients were divided into the high or low group based on the optimal cut-off, which was previously calculated using the same package. Kaplan-Meier analysis was employed to assess the two groups with the log-rank test. Afterwards, ROC analysis of time-independent outcomes was also performed.

### 2.5 Function Analysis

#### 2.5.1 Infiltrative Immune Cell Analysis

To characterise the abundance of 22 infiltrative immune cell types based on the expression matrix data of patients with GBM, the *CIBERSORTx* web tool (https://cibersortx.stanford.edu/) was applied (Newman et al., 2019). This tool uses batch correction to adjust the gene expression profile of the bulk of cells (mixture data) to eliminate possible cross-platform variations between the mixture data and the gene expression data of single cells (signature matrix) (Le et al., 2020). After enabling batch correction, performing the Bulk mode, and selecting the quantile normalisation algorithm, the absolute score for the proportion of 22 immune cell subsets in GBM samples was calculated. The samples with *p* < 0.05 were enrolled for further analysis because of the high reliability of the inferred results (Ali et al., 2016). Wilcoxon rank-sum test was used to compare the differences in the proportion of the 22 infiltrative immune cell subtypes between the high and low groups. The Spearman correlation test was used to further explore the correlation of the two indexes with immune cell types.

#### 2.5.2 Single Sample Gene Set Enrichment Analysis (ssGSEA)

The ssGSEA method, (Barbie et al., 2009), which is a modification of GSEA, (Subramanian et al., 2005), was developed to obtain an enrichment score for a single sample instead of two groups of samples. Here, the ssGSEA was used to compare differentially enriched hallmarks of cancer gene sets (Barbie et al., 2009). To identify key pathways in different groups, we chose to focus on 50 hallmark gene sets, which were designed to highlight gene sets contained in the Molecular Signatures Database (MSigDB), (Liberzon et al., 2015), one of the most widely used and comprehensive databases of gene sets for performing gene set enrichment analysis. The hallmarks of the gene sets effectively summarise most of the relevant information of the original founder sets and, by reducing both variation and redundancy, provide more refined and concise inputs for gene set enrichment analysis (Liberzon et al., 2015). Gene symbol profiles for *Homo sapiens* were downloaded from the MSigDB. Then, the degree of association between each hallmark’s ssGSEA profile was estimated using the *gsva* package. Next, differential analysis was performed with the *limma* package under the threshold of the absolute value of *t* > 1 and adjusted *p* value <0.05.

## 3 Results

### 3.1Patient Characteristics

**FIGURE 1:**
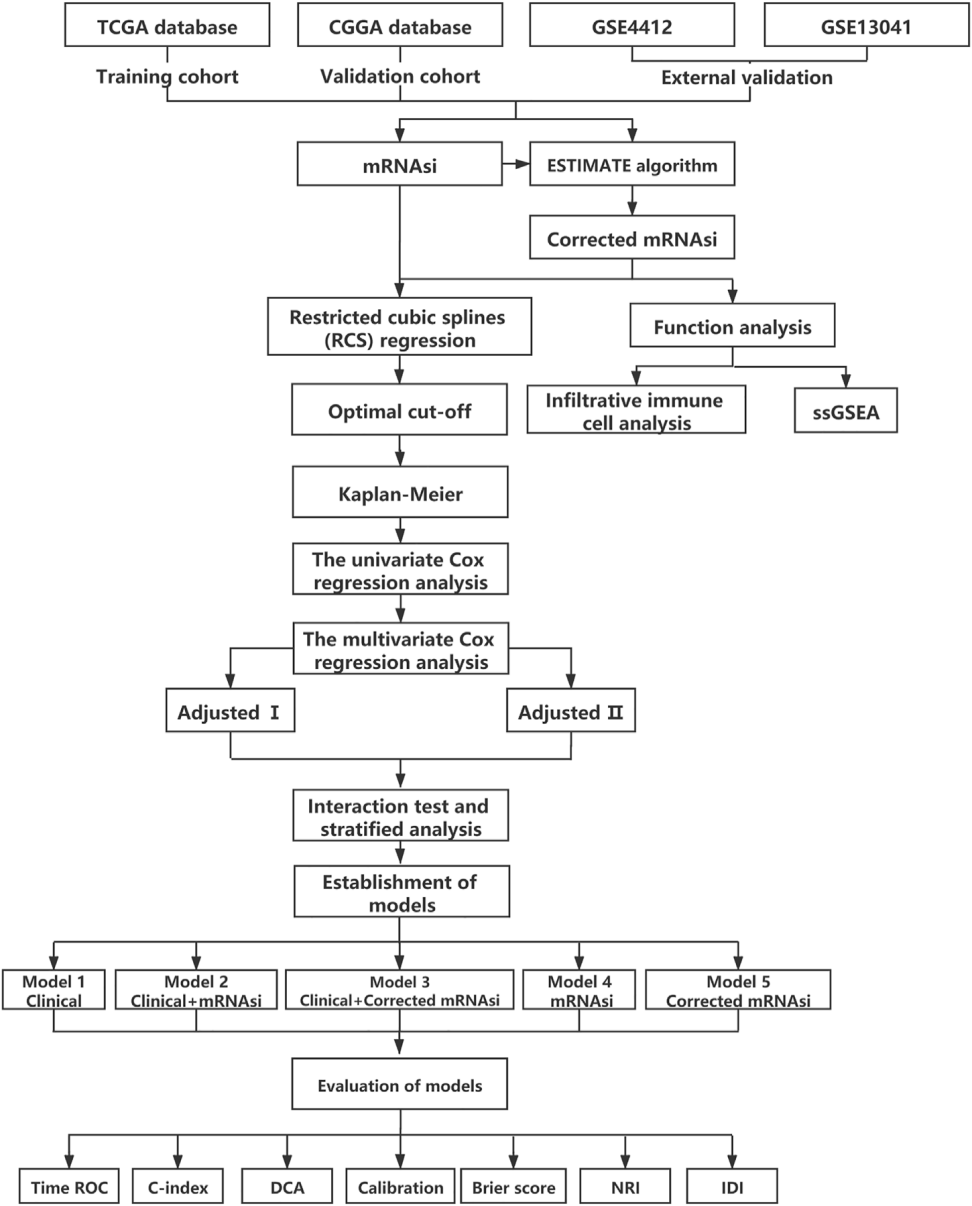
Flow chart.

An overview of the stemness indices-related signature development and validation workflow is presented in Figure 1. A total of 874 GBM samples (158 from TCGA as the training cohort, and 279 from CGGA and 437 from GEO as the validation cohort) were obtained in our study. The patient characteristics are presented in Table 1.

**TABLE 1:** Patient characteristics.

Character	Training cohort	External validation cohort	External validation GEO cohort	External validation GEO cohort
	TCGA (*n* = 158)	CGGA (*n* = 279)	GSE4412 (*n* = 170)	GSE13041 (*n* = 267)
Age	59.6 (13.6)	48.00 (39.5–57.0)	42.0 (33.0–54.0)	53.71 (13.8)
mRNAsi	0.56 (0.22)	0.50 (0.17)	0.60 (0.23)	0.42 (0.33–0.52)
c_mRNAsi	0.74 (0.25)	0.57 (0.16)	0.71 (0.25)	0.51 (0.15)
Male	102 (64.56%)	165 (59.14%)	64 (37.65%)	151 (63.18%)
IDH				
Wild type	148 (93.67%)	211 (75.63%)	NA	NA
Mutation	10 (6.33%)	68 (24.37%)	NA	NA
Radiotherapy				
No	29 (18.35%)	53 (19%)	NA	NA
Yes	129 (81.65%)	226 (81%)	NA	NA
Chemotherapy				
No	45 (28.48%)	49 (17.56%)	NA	NA
Yes	113 (71.52%)	230 (82.44%)	NA	NA

Data are presented as median (interquartile range) or N (%). TCGA, the cancer genome atlas; CGGA, chinese glioma genome atlas; GEO, gene expression omnibus; IDH, isocitrate dehydrogenase; NA, not applicable.

### 3.2 mRNAsi/c_mRNAsi Acts as an Independent Prognostic Factor

#### 3.2.1 Patients With High mRNAsi/c_mRNAsi GBM had a Better Prognosis

**FIGURE 2:**
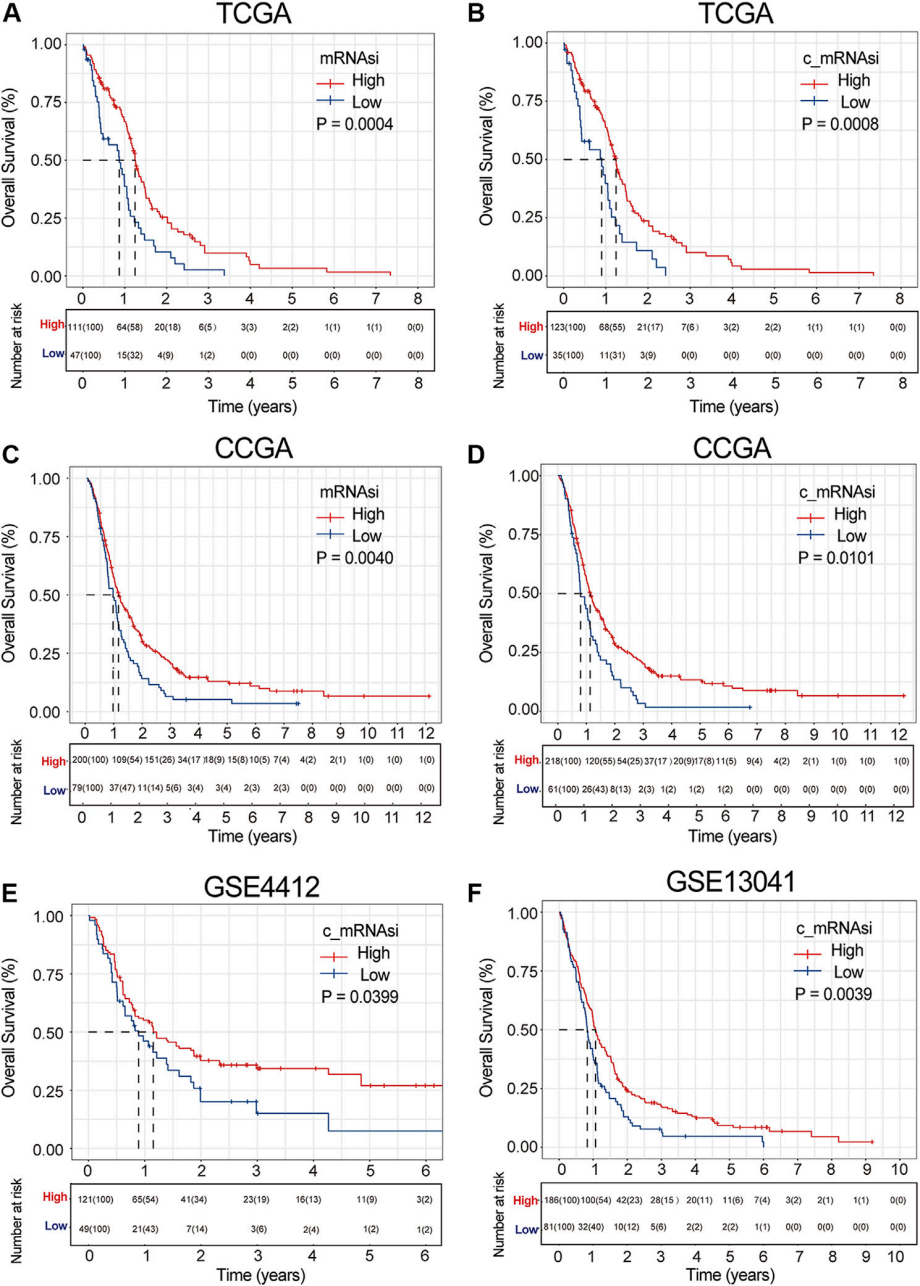
Survival curve of mRNAsi/c_mRNAsi on prognosis. **(A)**. Overall survival curve of mRNAsi in TCGA. **(B)**. Overall survival curve of c_mRNAsi in TCGA. **(C)**. Overall survival curve of mRNAsi in CGGA. **(D)**. Overall survival curve of c_mRNAsi in CGGA. **(E)**. Overall survival curve of c_mRNAsi in GSE4412. **(F)**. Overall survival curve of c_mRNAsi in GSE13041.

In the TCGA dataset, the relation between mRNAsi/c_mRNAsi and OS was nonlinear. Therefore, they were considered dichotomous variables in subsequent analysis (Supplementary Figures S1A,B). A total of 158 samples were clustered into the high- (*n* = 111) or low- (*n* = 47) mRNAsi group based on the optimal cut-off value identified by *survMisc* package (Supplementary Figure S2A). Patients in the high-mRNAsi group had better OS than those in the low-mRNAsi group (*p* = 0.0003) (Figure 2A). Similarly, 158 patients were clustered into the high- (*n* = 123) or low- (*n* = 35) c_mRNAsi group based on the optimal cut-off value identified by the same package (Supplementary Figure S2B). Patients in the high-c_mRNAsi group had better OS than those in the low-c_mRNAsi group (*p* = 0.0008) (Figure 2B). Moreover, we explored the relationship between mRNAsi/c_mRNAsi and disease-specific survival/progression-free interval in TCGA, and found that the trends for disease-specific survival (*p* = 0.0028) (Supplementary Figures S3A,B) and progression-free interval (*p* < 0.0001) (Supplementary Figures S3C,D) were similar to that for OS.

To determine whether the mRNAsi/c_mRNAsi-associated prognostic signature had a similar prognostic value in different populations, its prediction performance was validated externally in CGGA and GEO. Similarly, we considered mRNAsi/c_mRNAsi as a dichotomous variable in CGGA according to the Lowess result (Supplementary Figures S1C,D). All samples in CGGA and GEO were clustered into the high- or low-mRNAsi/c_mRNAsi group based on the optimal cut-off value identified by the same package (Supplementary Figure S2C). Consistent with the findings in TCGA, the Kalan-Meier curve in CGGA revealed that patients in the high-mRNAsi/c_mRNAsi group had better OS than those in the low-mRNAsi/c_mRNAsi group (*p* = 0.0040 and 0.0011, respectively) (Figures 2C,D). GSE4412 has the transcriptional profiling of 170 GBM samples from 74 patients (Freije et al., 2004). A total of 170 GBM samples were divided into the high- (*n* = 121) or low- (*n* = 49) c_mRNAsi group based on the optimal cut-off value (Supplementary Figure S2D), and the high-c_mRNAsi group had better OS (*p* = 0.0400) (Figure 2E). GSE13041 has 267 GBM samples from 239 patients, (Lee et al., 2008), which were divided into the high- (*n* = 186) or low- (*n* = 81) c_mRNAsi group based on the optimal cut-off value (Supplementary Figure S2). The high-c_mRNAsi group also had better OS (*p* = 0.0040) (Figure 2F).

#### 3.2.2 Identification of Confounding Variables

**FIGURE 3:**
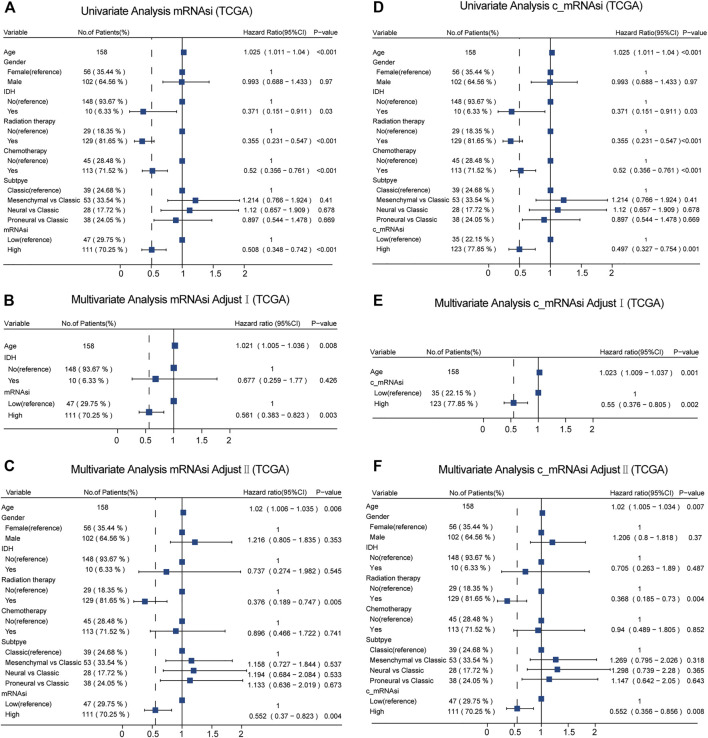
Forest plots of univariate and multivariate Cox regression analysis in TCGA. **(A)**. Univariate Cox regression analysis of mRNAsi in TCGA. **(B)**. Multivariate Cox regression analysis of mRNAsi adjusted I model in TCGA. **(C)**. Multivariate Cox regression analysis of mRNAsi adjusted II model in TCGA. **(D)**. Univariate Cox regression analysis of c_mRNAsi in TCGA. **(E)**. Multivariate Cox regression analysis of c_mRNAsi adjusted I model in TCGA. **(F)**. Multivariate Cox regression analysis of c_mRNAsi adjusted II model in TCGA.

Given the possible interference of confounding variables, we carried out confounders identification and then adjusted for these potential confounding factors. In TCGA, we found that mRNAsi had to be adjusted for age through univariate analysis (Figure 3A). These covariates combined with common covariates (age, gender, IDH, radiotherapy, chemotherapy, and subtype) were enrolled into the adjusted II model. In the adjusted I model, after adjusting for confounders (age and IDH), mRNAsi was still associated with OS (hazard ratio (HR) = 0.561, 95% confidence interval (CI) 0.383–0.823, *p* = 0.003) (Figure 3B). Furthermore, after adjusting for predominant clinical and prognostic factors (age, gender, IDH, radiotherapy, chemotherapy, and subtype) in the adjusted II model, mRNAsi independently predicted prognosis in TCGA (HR = 0.552, 95% CI 0.370–0.823, *p* = 0.004) (Figure 3C). The interaction analysis revealed that gender played an interactive role in the association between mRNAsi and OS (Supplementary Table S1). Male patients had higher HRs between mRNAsi and OS (HR = 0.92; 95% CI, 0.11–7.59) than females (HR = 0.32; 95% CI, 0.17–0.61). In the same way, we found that only age should be adjusted on c_mRNAsi through univariate analysis (Figure 3D), and this covariate combined with common covariates were enrolled in the adjusted II model. In the adjusted I model, after adjusting for the confounder (age), c_mRNAsi was still associated with OS (HR = 0.550, 95% CI 0.376–0.805, *p* = 0.002) (Figure 3E). Furthermore, after adjusting for predominant clinical and prognostic factors in the adjusted II model, c_mRNAsi independently predicted prognosis in TCGA (HR = 0.552, 95% CI 0.356–0.856, *p* = 0.008) (Figure 3F). The effect of mRNAsi/c_mRNAsi on OS was consistent across subgroups (Supplementary Table S1). Ultimately, mRNAsi/c_mRNAsi was an independent prognostic factor for OS in patients with GBM.

**FIGURE 4:**
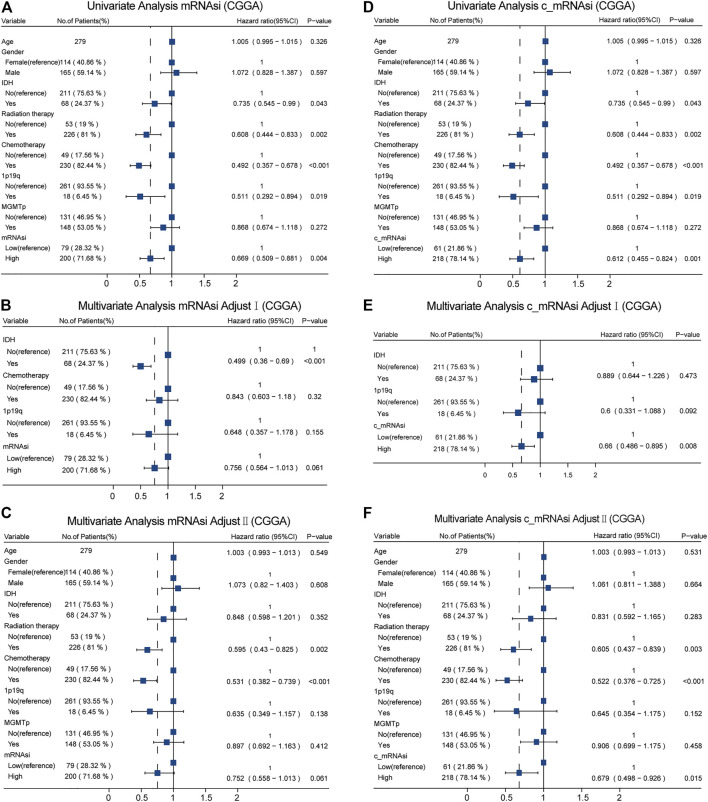
Forest plots of univariate and multivariate Cox regression analysis in CGGA. **(A)**. Univariate Cox regression analysis of mRNAsi in CGGA. **(B)**. Multivariate Cox regression analysis of mRNAsi adjusted I model in CGGA. **(C)**. Multivariate Cox regression analysis of mRNAsi adjusted II model in CGGA. **(D)**. Univariate Cox regression analysis of c_mRNAsi in CGGA. **(E)**. Multivariate Cox regression analysis of c_mRNAsi adjusted I model in CGGA. **(F)**. Multivariate Cox regression analysis of c_mRNAsi adjusted II model in CGGA.

Moreover, in CGGA, we identified different confounders (IDH, chemotherapy, and 1p19q on mRNAsi, as well as IDH and 1p19q on c_mRNAsi) that had to be adjusted through univariate analysis (Figures 4A,D), and these confounders (adjusted I model, Figures 4B,E) combined with common covariates (age, gender, IDH, radiotherapy, chemotherapy, 1p19q, and MGMTp) were enrolled in the adjusted II model (Figures 4C,F). We found that only c_mRNAsi was an independent prognostic signature in patients with GBM in both the adjusted I and adjusted II models (*p* = 0.008, 0.015, respectively) (Figures 4E,F) and across stratified analyses (Supplementary Table S2).

### 3.3 Construction and Evaluation of Prognostic Models

**FIGURE 5:**
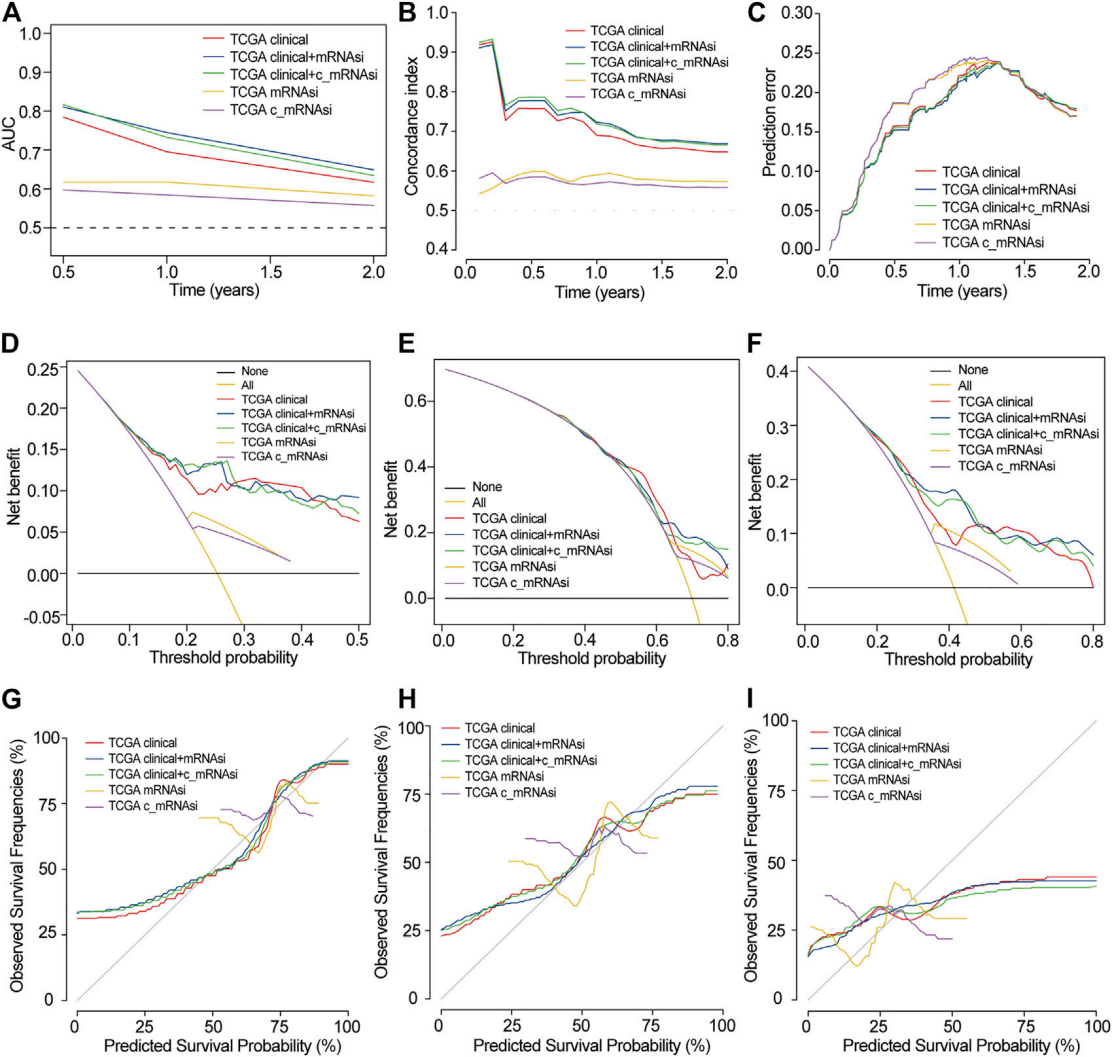
Evaluation of prognostic models in TCGA. **(A)**. AUC in TCGA. **(B)**. C-index in TCGA. **(C)**. Prediction error in TCGA. **(D)**. 0.5-years DCA in TCGA. **(E)**. 1-year DCA in TCGA. **(F)**. 1.5-years DCA in TCGA. **(G)**. 0.5-years calibration in TCGA. **(H)**. 1-year calibration in TCGA. **(I)**. 1.5-years calibration in TCGA.

We used discrimination, calibration, and model improvement capability to evaluate five established models. Models 2 and 3 had a higher area under the curve (AUC), better C-index, and lower prediction error than the other models (Figures 5A–C). The apparent and adjusted C-index as well as the Brier scores in years 0.5-, 1-, and 1.5-years indicated that models 2 and 3 were better than the others (Supplementary Table S3). DCA showed that the net benefit of models 2 and 3 in years 0.5 and 1 years was better than that of other models, but there was no significant difference in year 1.5 (Figures 5D–F). We found that the calibration of models 2 and 3 was better than that of other models in 0.5 and 1 years, while the calibration of the five models was poor in 1.5 years (Figures 5G–I). As for model improvement capability, when model 1 was considered as the reference, the NRI and IDI of models 2 and 3 were both positive. In contrast, the NRI and IDI of models 4 and 5 were both negative, although there were no significant statistical differences (Supplementary Table S4). From the above results, we determined that models 2 and 3 had good discrimination and calibration in the OS prediction of patients with GBM.

In CGGA, we also built five models that were similar to those in TCGA. In TCGA, only 24 patients were followed up for more than 2 years, and the prognosis was very poor. Since the median follow-up time was only 12 months, the selected time points were 0.5, 1 and 1.5 years. In CGGA, since the 62 cases were followed up for more than 2 years, the time point was extended to 3 and 5 years. Here, models 2 and 3 also had a higher AUC and better C-index but had the same prediction error as the other models (Supplementary Figures S4A–C). DCA showed that the net benefit of model 2 in 0.5, 1, 1.5, and 3 years was higher than that of other models, but there was no significant difference in 5 years (Supplementary Figures S4D–H). The calibration of models 2 and 3 was better than that of other models in 0.5 and 1 years, while the calibration of the five models was poor in 1.5, 3, and 5 years (Supplementary Figures S4I–M). The Brier scores indicated that models 2 and 3 were better than the others (Supplementary Table S5). As for model improvement capability, when model 1 was considered as the reference, the NRI and IDI of models 2 and 3 were both positive. In contrast, the NRI and IDI of models 4 and 5 were both negative (Supplementary Table S6). Because of the limited clinical data of GEO, we only compared models 4 and 5. The AUCs were 0.536–0.618, 0.544–0.589 in GSE4412 and GSE13041, respectively (Supplementary Figures S4N,O).

Combined with the above results, we found that mRNAsi/c_mRNAsi is an independent prognostic factor in TCGA, but only c_mRNAsi is an independent prognostic factor in CGGA. In TCGA, the comparison of the five models revealed that models 2 and 3 were the best, and there was little difference between these two models. In CGGA, model 3 performed the best among the five models. In GEO, there was no significant difference between the single mRNAsi and c_mRNAsi models. Therefore, we finally decided to adopt model 3 (clinical factors integrated with the c_mRNAsi) to predict OS and construct a nomogram in TCGA (Supplementary Figure S4). According to the nomogram, a representative patient with the total point of 286, the 0.5-years, 1-year, and 1.5-years survival rates were 82.6, 68.9, and 40.4%, respectively (Supplementary Figure S5).

### 3.4 Functional Analysis

#### 3.4.1 Differential Abundance of Infiltrative Immune Cells

**FIGURE 6:**
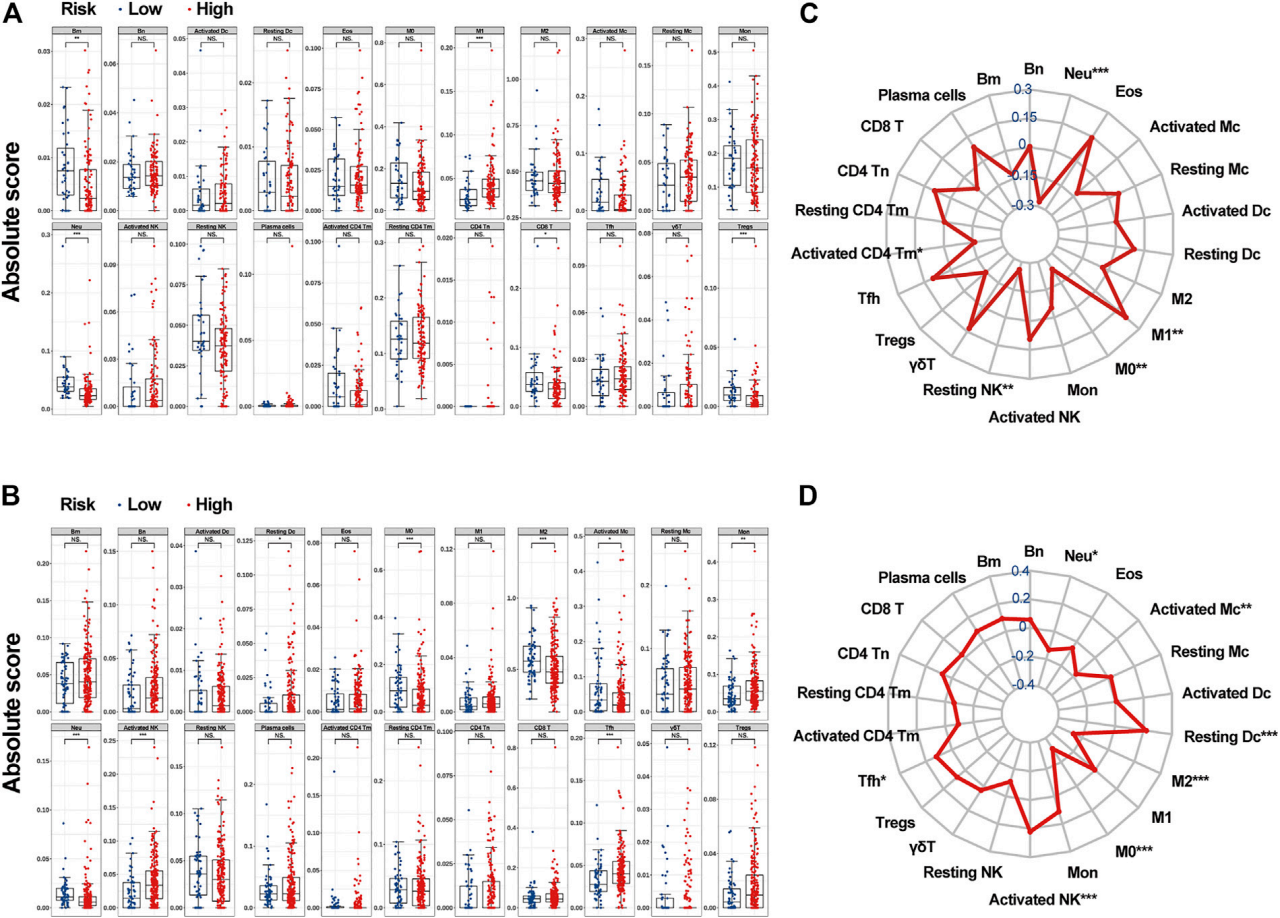
The associations between c_mRNAsi and the abundance of infiltrative immune cells. **(A)**. Infiltrative immune cell analysis in TCGA. **(B)**. Infiltrative immune cell analysis in CGGA. **(C)**. Radar chart in TCGA. **(D)**. Radar chart in CGGA. Abbreviations: Bm, memory B cells; Bn, naive B cells; Dc, Dendritic cells; Eos, eosinophils; M0, macrophage M0; M1, macrophage M1; M2, macrophage M2; Mc, mast cells; Mon, monocytes; Neu, neutrophils; Tm, memory T cells; Tn, naïve T cells; Tfh, follicular helper T cells; γδT, gamma delta T cells; Treg, regulatory T cells.

By applying the *CIBERSORTx* algorithm, the relative proportions of 22 immune cell subsets in GBM were acquired. A total of 158 patients with GBM from TCGA and 279 patients with GBM from the CGGA were enrolled for further analysis. In the TCGA dataset, the infiltration level of M1 macrophages was significantly higher in the high-c_mRNAsi group, whereas the infiltration levels of memory B cells, neutrophils, CD8^+^ T cells, and regulatory T cells were significantly higher in the low-c_mRNAsi group (Figure 6A). In CGGA, the infiltration levels of resting dendritic cells, monocytes, activated NK cells, and follicular T helper cells were significantly higher in the high-c_mRNAsi group, whereas macrophages (M0 and M2), activated mast cells, and neutrophils were significantly higher in the low-c_mRNAsi group (Figure 6B). Radar chart indicated that in the TCGA dataset, c_mRNAsi was positively correlated with M1 macrophages and negatively correlated with neutrophils, M0 macrophages, resting NK cells, and activated memory CD4^+^ T cells in the training cohort (Figure 6C). In the CGGA dataset, c_mRNAsi was positively correlated with resting dendritic cells, activated NK cells, and follicular T helper cells, and negatively correlated with neutrophils, activated mast cells, and macrophages (M0 and M2) (Figure 6D).

#### 3.4.2 Pathway Enrichment Analysis

**FIGURE 7:**
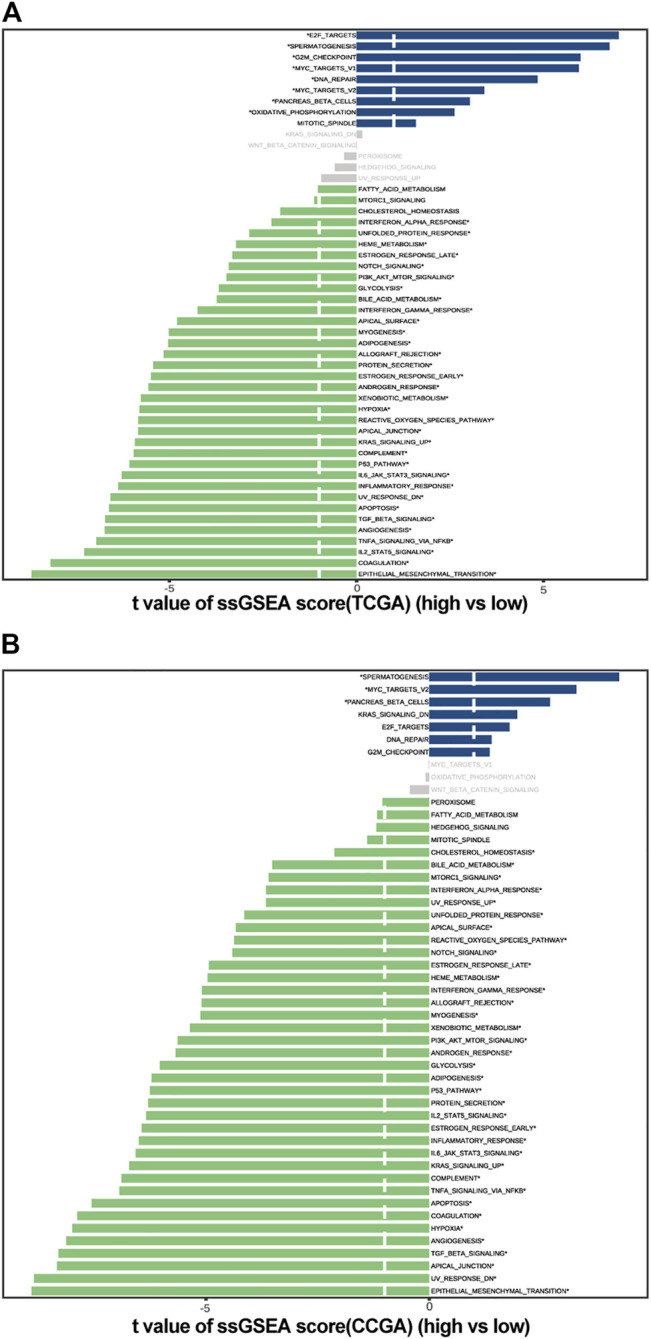
Pathway enrichment analysis in high and low c_mRNAsi groups. **(A)**. Pathway enrichment analysis in TCGA. **(B)**. Pathway enrichment analysis in CGGA.

The ssGSEA was used to estimate the degree of enrichment of the MSigDB hallmark gene set in individual samples from the high- and low-c_mRNAsi groups of both TCGA and CGGA. This allowed us to identify signalling pathways involved in GBM and to estimate their degree of association with each group (high versus low). The results indicated that spermatogenesis, MYC targets v2, and pancreas beta cell pathways were involved significantly in the low-c_mRNAsi group of both TCGA and CGGA, whereas unfolded protein response, haem metabolism, early and late oestrogen response, NOTCH signalling, glycolysis, bile acid metabolism, interferon α/γ response, apical surface, myogenesis, adipogenesis, allograft rejection, androgen response, xenobiotic metabolism, hypoxia, reactive oxygen species pathway, apical junction, KRAS signalling, complement, P53, IL6/JAK/STAT3 signalling, inflammatory response, UV response DN, apoptosis, TGF-β signalling, angiogenesis, TNFα signalling via NF-κB, IL-2/STAT5 signalling, coagulation, and epithelial mesenchymal transition pathways were involved significantly in the high-c_mRNAsi group of both TCGA and CGGA (Figures 7A,B).

## 4 Discussion

GBM is composed of non-homogeneous cell populations exhibiting varying degrees of genetic and functional heterogeneity. Cancer stem cells are capable of sustaining tumours by manipulating genetic and non-genetic factors to metastasise, resist treatment, and maintain the tumour microenvironment (Saygin et al., 2019). Understanding the key traits and mechanisms of stemness of cancer stem-like cells provides opportunities to improve patient outcomes via improved prognostic models and therapeutics. However, tumour cells are usually comprised of a heterogeneous mixture of subclones, each of which may have its own distinct characteristics. Therefore, accurately assessing the make-up of the different cell states within a tumour biopsy is very important. Here, we calculated an mRNAsi, which was also corrected by tumour purity (c_mRNAsi), based on the expression profile of 12,953 genes in 874 GBM samples from the TCGA, CGGA, and GEO public databases using the OCLR machine-learning algorithm. We found that, after confounding variable identification and interaction and stratified analyses, c_mRNAsi remained an independent prognostic factor in both TCGA and CGGA, whereas mRNAsi was affected by gender in TCGA and was no longer an independent prognostic factor after adjustment in CGGA. Model 2 (clinical factors integrated with mRNAsi) and model 3 (clinical factors integrated with c_mRNAsi) showed better calibration and discrimination than clinical factors alone in both TCGA and CGGA. Moreover, model 3 performed better than model 2, although there was no significant difference between the single mRNAsi and c_mRNAsi models in GEO. Therefore, we concluded that c_mRNAsi can be used as a new index for the construction of algorithms that predict the prognosis of patients with GBM. To explore the possible reasons for the difference in prognosis between the high- and low-c_mRNAsi groups, we applied the *CIBERSORTx* algorithm to infer the abundance of immune infiltrating cells in TCGA and CGGA and found differential infiltration patterns across 5 and 8 clusters in TCGA and CGGA, respectively. Importantly, we found that high mRNAsi correlated significantly with high infiltration of immune activated cells, especially M1 macrophages, dendritic cells, monocytes, activated NK cells, and follicular T helper cells. Lastly, we screened the potential signalling pathways of the c_mRNAsi-related signature and found that most of the pathways were immune-related.

In this study, we utilised the OCLR machine-learning algorithm to quantify mRNAsi and c_mRNAsi for each GBM sample. Using OCLR, we previously identified undiscovered biological mechanisms associated with the dedifferentiated oncogenic state (Lian et al., 2019). Moreover, OCLR exhibited comparable performance with a more flexible and convenient formulation to that with traditional support vector machine-based one-class predictors (Sokolov et al., 2016). We drew support from OCLR machine-learning algorithm to derive two distinct molecular metrics of stemness indices and finally selected c_mRNAsi for subsequent validation analysis, owing to its observed prognostic significance in various data. Stemness indices had already been identified in several malignancies and had different prognostic values in ovarian cancer, (Kaipio et al., 2020), medulloblastoma, (Lian et al., 2019), colon cancer, (Tao et al., 2019), or acute myeloid leukaemia (Seneviratne et al., 2019). However, stemness indices are targeted at bulk tissues, a mixture of tumour tissue and normal tissue. Although some scholars developed new algorithms to adjust stemness indices, (Pan et al., 2019), the algorithms are complex. The ESTIMATE algorithm can adjust directly from transcriptome data, (Yoshihara et al., 2013), which is more convenient. In our multi-cohort screening, we examined the capacity of c_mRNAsi, which is obtained after correcting the index by the tumour purity calculated with ESTIMATE, (Yoshihara et al., 2013), to predict OS. Our findings demonstrate that c_mRNAsi can be implemented in clinical practice, something that has not been previously reported. Common clinical indicators of GBM include KPS, *MGMT*, *IDH1*, and epidermal growth factor receptor vIII (Burgenske et al., 2019; Chaddad et al., 2019). GBM-specific microRNAs, including miR-21 and miR-10b, have also been presented as biomarkers with promising prognostic values (Sasmita et al., 2018). Confounder identification and interaction tests could help us to better understand the relationship between the variables and disease. In our study, we first used the mRNAsi/c_mRNAsi calculated by the algorithm as a variable to carry out residual confounder identification and interaction test with common clinical indicators, so as to minimise the impact of confounding factors on GBM OS as much as possible. Furthermore, discrimination and calibration are the most commonly used indicators in the evaluation of clinical prediction models. However, a systematic review found that while 63% of the studies on prediction models reported discrimination data, only 36% included calibration data (Wessler et al., 2015). In the present study, we report both discrimination and calibration in the training and validation cohorts. In addition, we did not only use the enhanced bootstrap test for internal validation, but also directly compared multiple models in two data sets (TCGA and CGGA) to minimise model overfitting. This significantly differs from traditional studies of clinical models, and allowed us to select the optimal model for prognosis prediction in patients with GBM.

Previous studies have shown that the higher the stemness indices scores, the worse the overall survival outcomes, (Pei et al., 2020), which is the opposite from our results. Therefore, we wanted to further explore the reasons for the different prognosis among the groups. Using gene-expression-based metrics, a recent study reported the association of stemness with immune cell infiltration and genomic, transcriptomic, and clinical parameters across 21 solid cancers (Miranda et al., 2019). Pervasive negative associations between cancer stemness and anticancer immunity have also been found (Miranda et al., 2019). In line with the current pan-cancer findings, we also analysed infiltrative immune cells in distinct cohorts (TCGA and CGGA) using the *CIBERSORTx* algorithm and found that the high-c_mRNAsi group exhibited significant immune suppression. Based on the expression data in TCGA and CGGA, we observed that c_mRNAsi correlated negatively with infiltrating levels of immune cells. Promoting Treg overrepresentation and function induces systemic and intratumoural immunosuppression (Long et al., 2020). CD8^+^ cytotoxic T lymphocyte cells, macrophages, Tregs, and other immune cells can respond to GBM treatment, including immunotherapy, to a certain extent (Choi et al., 2019). Meanwhile, high c_mRNAsi was associated with the up-regulation of M1 macrophages, resting dendritic cells, monocytes, activated NK cells, and follicular T helper cells. In addition, we also found that general immune-related pathways were activated in the high-c_mRNAsi group, which is consistent with previous findings (Zhang C. et al., 2020). Collectively, our results suggest that the better prognosis of patients with high c_mRNAsi may be owing to the presence of more tumour stem cells and more tumour neoantigens, which results in the higher infiltration of tumour immune cells. Based on our findings, we propose c_mRNAsi as a new marker for tumour immunotherapy in the future.

The stemness indices reflects the ability of self-renewal and unlimited proliferation. We found that significantly different enrichment pathways are mainly related to cell cycle, damage repair, proliferation, apoptosis, angiogenesis, glucose, lipid metabolism and energy metabolism through ssGSEA. The current view is that tumour stem cells are related to the inhibitory immune microenvironment (Alves et al., 2021). At present, it is found that the stemness indices is also related to IL6/JAK/STAT3, IL-2/STAT5, and TGF-β signalling pathways (Zhang et al., 2019; Liu et al., 2021; Mo et al., 2021; Zhou et al., 2021). Interleukins are closely related to the proliferation and function of T cells (Ceuppens et al., 1988; Popmihajlov et al., 2012; Raeber et al., 2018). Tregs are a key source of TGF-β ligands (Zhang Y. et al., 2020). Together, the pathways we enriched here are closely related to immune response.

There are several limitations in our study that need to be addressed in the future. First, we used four different datasets (TCGA, CGGA, GSE4412, and GSE13041) to test the prognostic value of mRNAsi/c_mRNAsi and found the power and robustness of c_mRNAsi. However, we could not definitively determine whether the c_mRNAsi obtained from the bulk tumour sequencing/array could be utilised for all types of GBM samples from diverse genetic backgrounds. Furthermore, the study was based on public data. We should use our own sequencing data to verify the c_mRNAsi and clinical model. Besides, the c_mRNAsi signature could distinguish differential subpopulations with distinct prognosis. Whether stemness indices mediate poor immunotherapy response requires further investigation. There is still a long way before we can accomplish individualised classifications for treatment because other clinical and genetic/epigenetic factors must be considered and incorporated into treatment decision-making (Mansouri et al., 2019). In addition, due to the limitation of GEO data sources, we could only verify the prognostic signature of c_mRNAsi, but could not identify confounding factors as in CGGA. Moreover, the c_mRNAsi-related signature should be further validated in large samples of patients with GBM from multiple centres to identify the associations not only with survival outcomes but also conventional drug responses, especially immunotherapy. Lastly, although we performed functional analysis and identified numerous differences in infiltrative immune cell abundance and the regulation of related pathways, specific experimental validations need to be designed to assess the real effect. Despite the above shortcomings, our work has certain advantages that cannot be ignored. We calculated the mRNAsi/c_mRNAsi using a large number of samples and, for the first time, performed confounding variable identification and interaction and stratified analyses. Furthermore, we made a comprehensive comparison of several models, and proved the validity of our conclusions in multiple ways to verify the credibility of our results.

In conclusion, our study systematically assessed the GBM stemness indices based on multiple independent cohorts, providing a robust quantified mRNAsi/c_mRNAsi reflective of stemness indices, and the associations with immune infiltration and immune related pathways. The c_mRNAsi-based signature proved to be superior to other models in predicting OS prognosis, and may be a valuable classifier for uncovering distinct subgroups of stemness indices.

## Data Availability

The datasets presented in this study can be found in online repositories. The names of the repository/repositories and accession number(s) can be found in the article/Supplementary Material.

## Supplementary Material

Supplementary Figure 1 Lowess results. **(A)**. mRNAsi with OS in TCGA. **(B)**. c_mRNAsi with OS in TCGA. **(C)**. mRNAsi with OS in CGGA. **(D)**. c_mRNAsi with OS in CGGA.

Supplementary Figure 2 Identification of optimal cut-off values. **(A)**. Optimal cut-off value of mRNAsi in TCGA. **(B)**. Optimal cut-off value of c_mRNAsi in TCGA. **(C)**. Optimal cut-off value of mRNAsi in CGGA. **(D)**. Optimal cut-off value of c_mRNAsi in CGGA.

Supplementary Figure 3 Survival curve of mRNAsi/c_mRNAsi on prognosis. **(A)**. Disease-specific survival curve of mRNAsi in TCGA. **(B)**. Disease-specific survival curve of c_mRNAsi in TCGA. **(C)**. Progression-free interval curve of mRNAsi in TCGA. **(D)**. Progression-free interval curve of c_mRNAsi in TCGA.

Supplementary Figure 4 External validation in CGGA and GEO. **(A)**. AUC in TCGA. **(B)**. C-index in CGGA. **(C)**. Prediction error in CGGA. **(D)**. 0.5-year DCA in CGGA. **(E)**. 1-year DCA in CGGA. **(F)**. 1.5-year DCA in CGGA. **(G)**. 3-year DCA in CGGA. **(H)**. 5-year DCA in CGGA. **(I)**. 0.5-year calibration in CGGA. (J). 1-year calibration in CGGA. **(K)**. 1.5-year calibration in CGGA. **(L)**. 3-year calibration in CGGA. **(M)**. 5-year calibration in CGGA. **(N)**. AUC in GSE4412. **(O)**. AUC in GSE13041.

Supplementary Figure 5 Construction of prognostic nomogram.
