# Supplementary figures and images for: Prognostic Value of mRNAsi/Corrected mRNAsi Calculated by the One-Class Logistic Regression Machine-Learning Algorithm in Glioblastoma Within Multiple Datasets
Source: Front Mol Biosci. 2021 Dec 6;8:777921. doi: 10.3389/fmolb.2021.777921 (PMC8685528; doi:10.3389/fmolb.2021.777921)

**A****TCGA**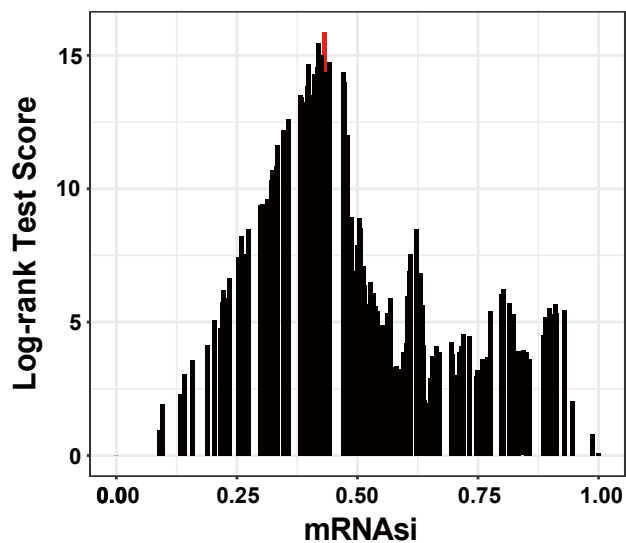**B****TCGA**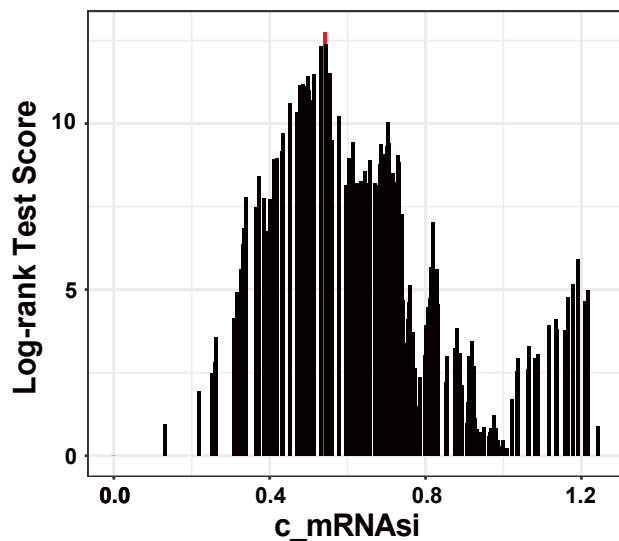**C****CGGA**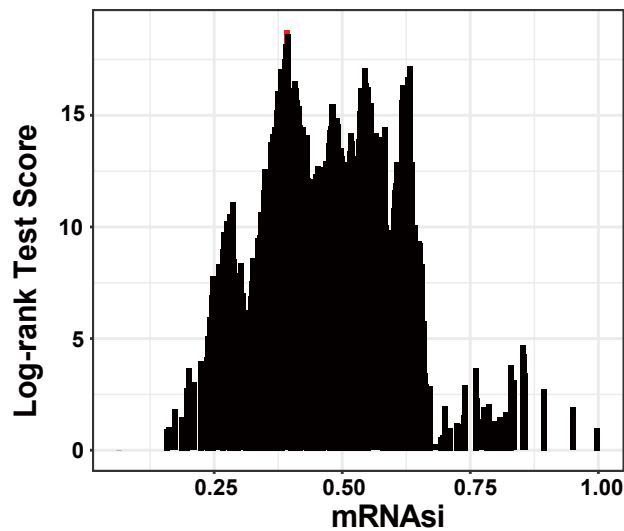**D****CGGA**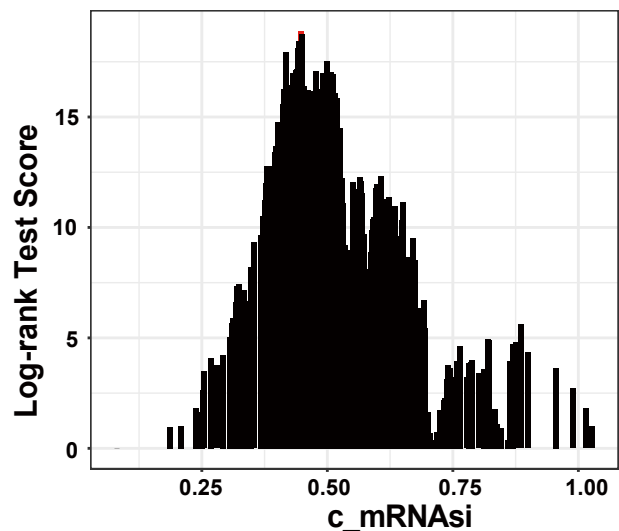

Supplement: Supplementary file 1 [file DataSheet2.PDF]

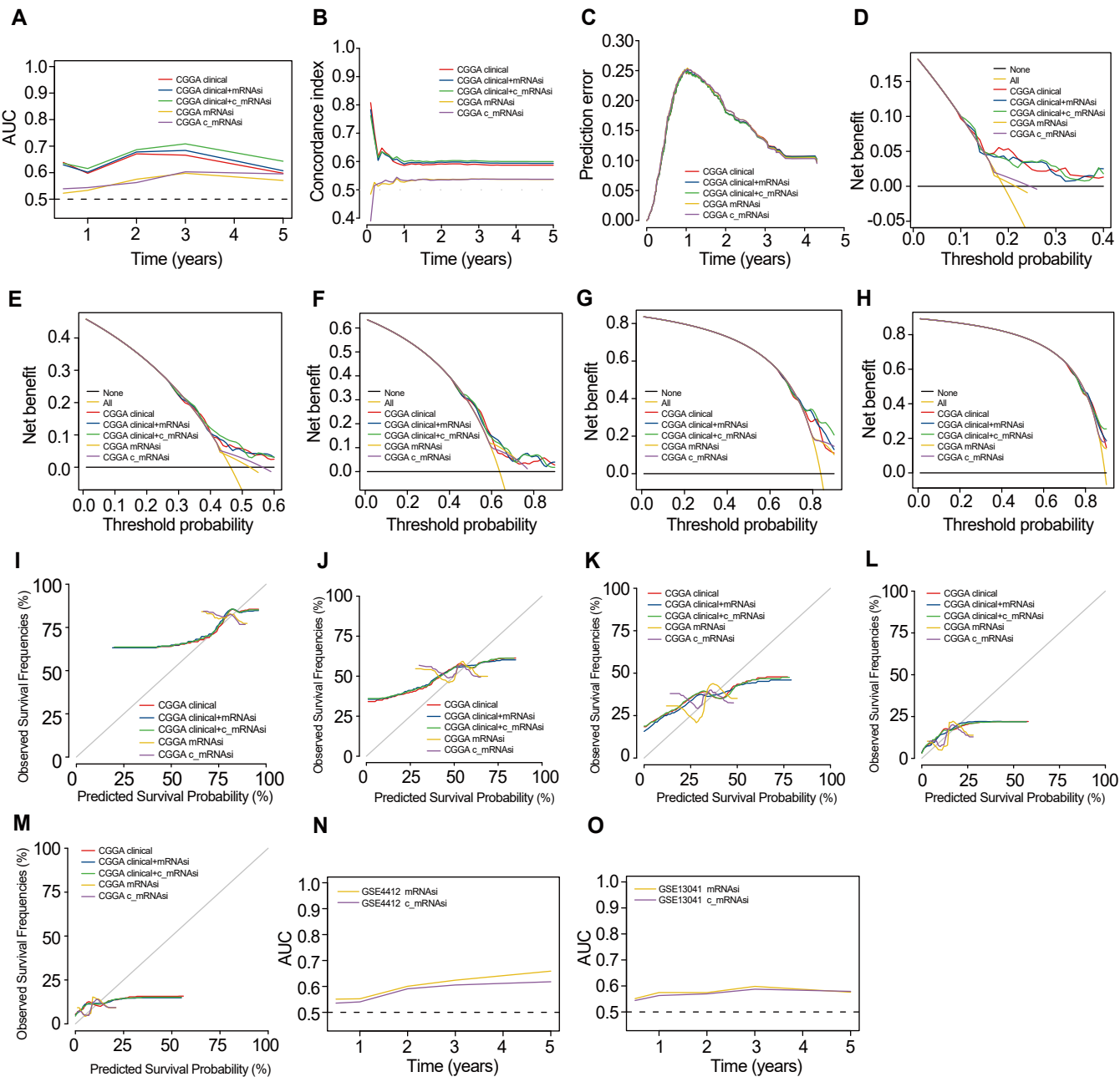

Supplement: Supplementary file 2 [file DataSheet4.PDF]

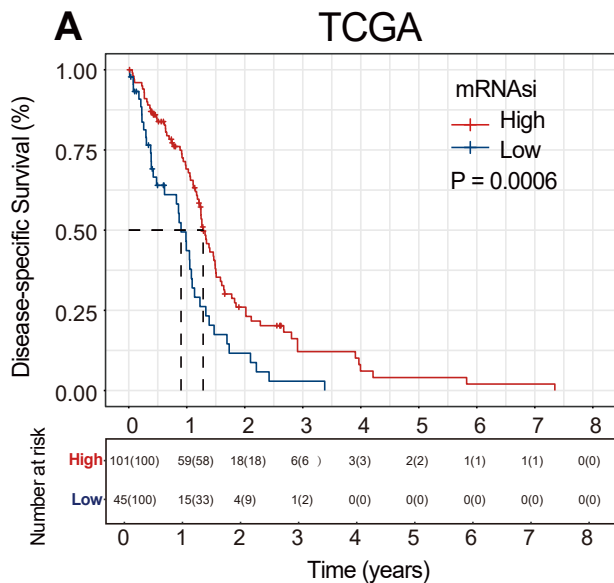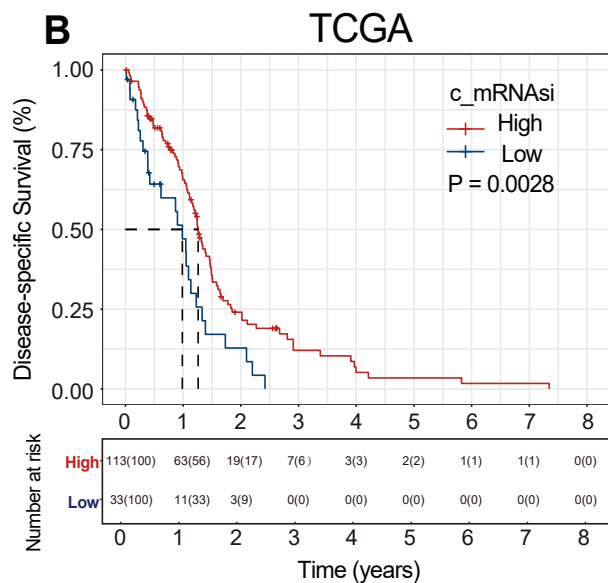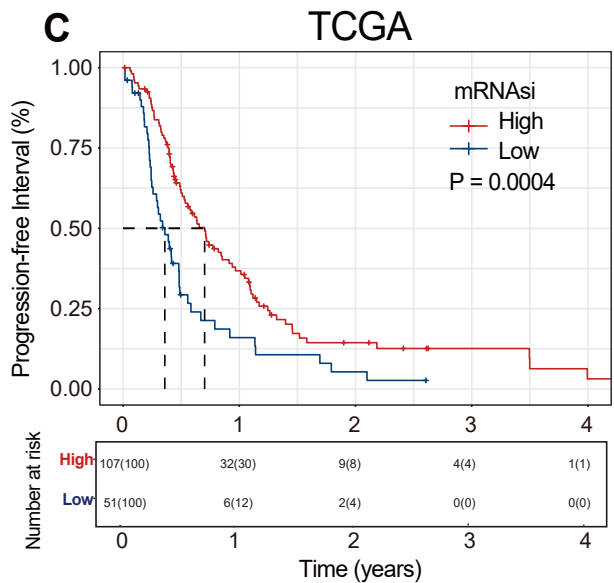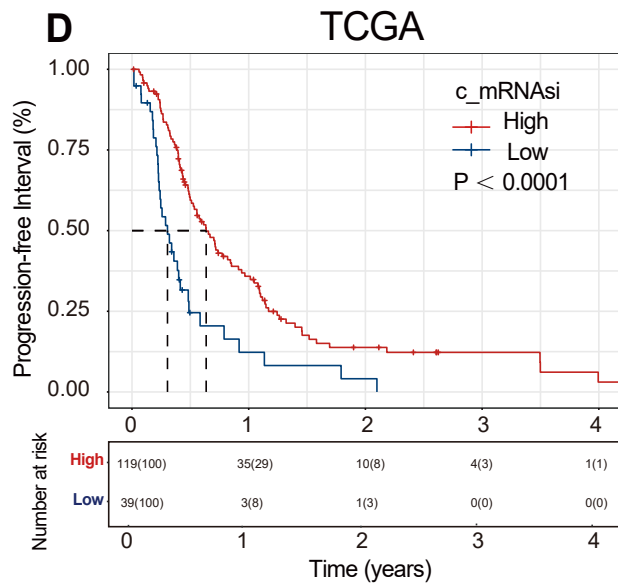

Supplement: Supplementary file 4 [file DataSheet3.PDF]

**A****TCGA**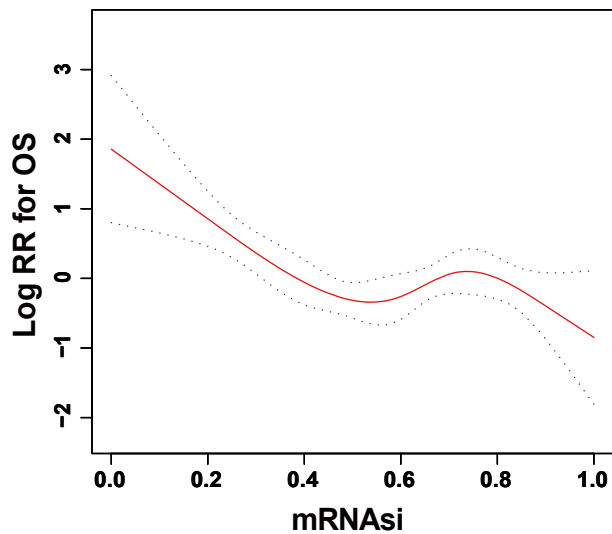**B****TCGA**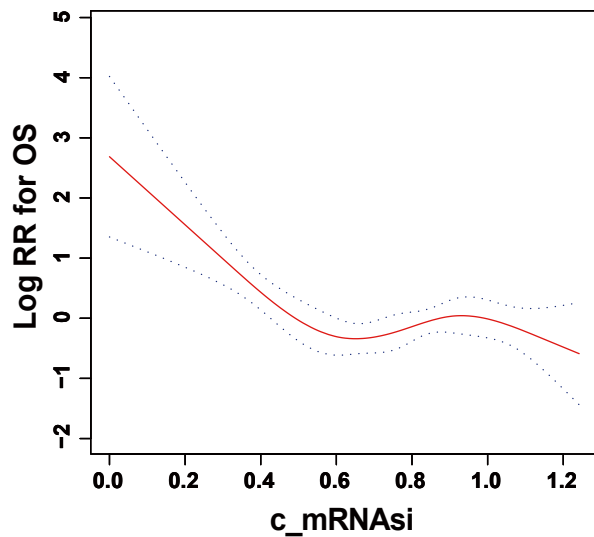**C****CGGA**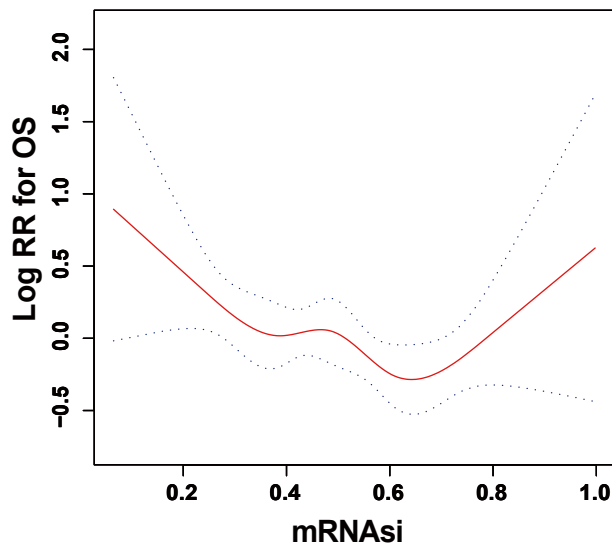**D****CGGA**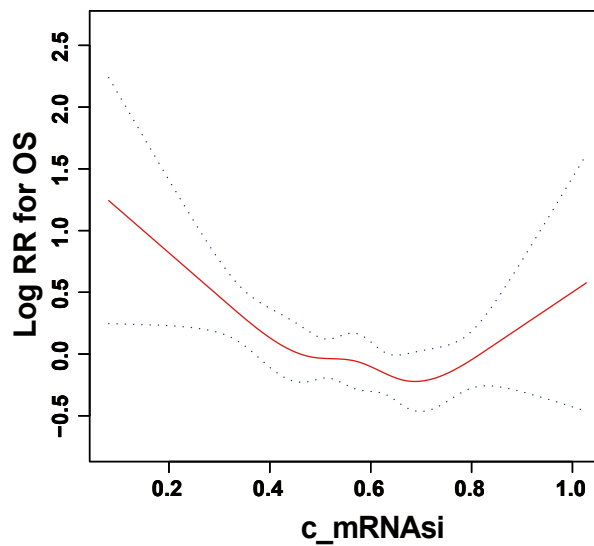

Supplement: Supplementary file 6 [file DataSheet1.PDF]

Nomogram (Clinical+c\_mRNAsi)

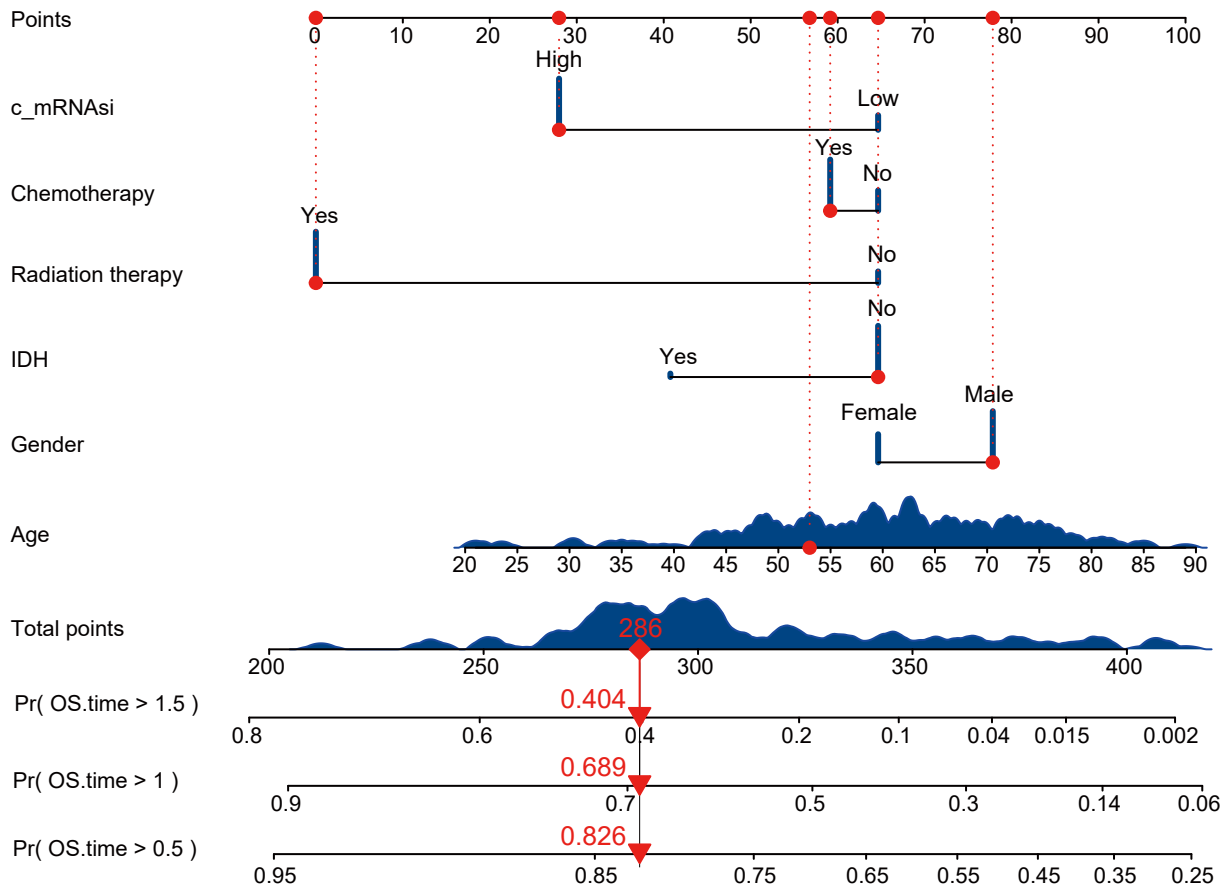

Supplement: Supplementary file 8 [file DataSheet5.PDF]
