# Supplementary material for: Prognostic Value of mRNAsi/Corrected mRNAsi Calculated by the One-Class Logistic Regression Machine-Learning Algorithm in Glioblastoma Within Multiple Datasets
Source: Front Mol Biosci. 2021 Dec 6;8:777921. doi: 10.3389/fmolb.2021.777921 (PMC8685528; doi:10.3389/fmolb.2021.777921)
Supplement: Supplementary file 3 [file Table1.DOCX]

Table S1. Interaction test and stratified analysis in TCGA

|  | mRNAsi | | | | | | c_mRNAsi | | | | | |
| --- | --- | --- | --- | --- | --- | --- | --- | --- | --- | --- | --- | --- |
|  | Group | n | HR (95% CI) | *P* for interaction | ^1^HR (95% CI) | *P* for interaction | Group | n | HR (95% CI) | *P* for interaction | 1HR (95% CI) | *P* for interaction |
| Age |  |  |  | 0.8218 |  |  |  |  |  | 0.409 |  |  |
| <54y | Low | 12 | 1.0 (ref.) |  |  |  | Low | 9 | 1.0 (ref.) |  |  |  |
| <54y | High | 38 | 0.43(0.2-0.93) a |  |  |  | High | 41 | 0.42(0.18-0.97) a |  |  |  |
| 54y-65y | Low | 16 | 1.07(0.46-2.44) |  |  |  | Low | 10 | 1.46(0.54-3.96) |  |  |  |
| 54y-65y | High | 37 | 0.61(0.29-1.28) |  |  |  | High | 43 | 0.58(0.26-1.32) |  |  |  |
| >65y | Low | 19 | 1.38(0.61-3.11) |  |  |  | Low | 16 | 1.12(0.45-2.79) |  |  |  |
| >65y | High | 36 | 0.8(0.38-1.68) |  |  |  | High | 39 | 0.81(0.36-1.84) |  |  |  |
| Total | Low | 47 | 1.0 (ref.) |  |  |  | Low | 35 | 1.0 (ref.) |  |  |  |
| Total | High | 111 | 0.54(0.37-0.79) b |  |  |  | High | 123 | 0.54(0.35-0.82) b |  |  |  |
| Gender |  |  |  | 0.039 |  | 0.043 |  |  |  | 0.268 |  | 0.371 |
| Female | Low | 19 | 1.0 (ref.) |  | 1.0 (ref.) |  | Low | 13 | 1.0 (ref.) |  | 1.0 (ref.) |  |
| Female | High | 37 | 0.29(0.15-0.54) c |  | 0.32(0.17-0.61) c |  | High | 43 | 0.35(0.17-0.71) b |  | 0.41(0.2-0.87) a |  |
| Male | Low | 28 | 0.52(0.28-0.99) a |  | 1.26(0.15-10.72) |  | Low | 22 | 0.63(0.29-1.36) |  | 1.98(0.23-17.4) |  |
| Male | High | 74 | 0.35(0.2-0.61) c |  | 0.92(0.11-7.59) |  | High | 80 | 0.36(0.18-0.71) b |  | 1.25(0.15-10.1) |  |
| Total | Low | 47 | 1.0 (ref.) |  | 1.0 (ref.) |  | Low | 35 | 1.0 (ref.) |  | 1.0 (ref.) |  |
| Total | High | 111 | 0.5(0.34-0.74) c |  | 0.56(0.38-0.82) b |  | High | 123 | 0.49(0.32-0.75) b |  | 0.55(0.36-0.85) b |  |
| Radiotherapy |  |  |  | 0.096 |  | 0.436 |  |  |  | 0.268 |  | 0.961 |
| No | Low | 13 | 1.0 (ref.) |  | 1.0 (ref.) |  | Low | 10 | 1.0 (ref.) |  | 1.0 (ref.) |  |
| No | High | 16 | 0.26(0.12-0.59) b |  | 0.41(0.18-0.97) a |  | High | 19 | 0.31(0.13-0.74) b |  | 0.55(0.22-1.36) |  |
| Yes | Low | 34 | 0.2(0.1-0.42) c |  | 0.95(0.1-8.67) |  | Low | 25 | 0.23(0.1-0.53) c |  | 1.12(0.12-10.88) |  |
| Yes | High | 95 | 0.12(0.06-0.23) c |  | 0.57(0.06-5.11) |  | High | 104 | 0.13(0.06-0.27) c |  | 0.63(0.07-5.7) |  |
| Total | Low | 47 | 1.0 (ref.) |  | 1.0 (ref.) |  | Low | 35 | 1.0 (ref.) |  | 1.0 (ref.) |  |
| Total | High | 111 | 0.49(0.34-0.72) c |  | 0.56(0.38-0.83) b |  | High | 123 | 0.49(0.32-0.74) c |  | 0.56(0.36-0.86) b |  |
| Chemotherapy |  |  |  | 0.171 |  | 0.492 |  |  |  | 0.463 |  | 0.911 |
| No | Low | 17 | 1.0 (ref.) |  | 1.0 (ref.) |  | Low | 13 | 1.0 (ref.) |  | 1.0 (ref.) |  |
| No | High | 28 | 0.31(0.16-0.63) b |  | 0.44(0.21-0.92) a |  | High | 32 | 0.38(0.18-0.81) b |  | 0.58(0.26-1.27) |  |
| Yes | Low | 30 | 0.32(0.16-0.64) b |  | 1.34(0.15-12.11) |  | Low | 22 | 0.39(0.18-0.86) b |  | 1.67(0.18-15.67) |  |
| Yes | High | 83 | 0.18(0.1-0.34) c |  | 0.8(0.09-7.05) |  | High | 91 | 0.21(0.11-0.42) c |  | 0.91(0.1-7.98) |  |
| Total | Low | 47 | 1.0 (ref.) |  | 1.0 (ref.) |  | Low | 35 | 1.0 (ref.) |  | 1.0 (ref.) |  |
| Total | High | 111 | 0.48(0.33-0.7) c |  | 0.55(0.37-0.81) b |  | High | 123 | 0.49(0.32-0.74) c |  | 0.56(0.36-0.85) b |  |
| GeneExp_Subtype |  |  |  | 0.490 |  | 0.293 |  |  |  | 0.818 |  | 0.823 |
| Classic | Low | 9 | 1.0 (ref.) |  | 1.0 (ref.) |  | Low | 9 | 1.0 (ref.) |  | 1.0 (ref.) |  |
| Classic | High | 30 | 0.68(0.31-1.53) |  | 0.6(0.25-1.41) |  | High | 30 | 0.69(0.31-1.53) |  | 0.6(0.26-1.42) |  |
| Mesenchymal | Low | 29 | 1.58(0.7-3.52) |  | 0.27(0.01-8.06) |  | Low | 18 | 1.59(0.66-3.82) |  | 0.46(0.02-13.59) |  |
| Mesenchymal | High | 24 | 0.59(0.26-1.35) |  | 0.08(0-2.71) |  | High | 35 | 0.75(0.34-1.64) |  | 0.2(0.01-6.01) |  |
| Neural | Low | 7 | 1.01(0.36-2.82) |  | 0.01(0-0.28) b |  | Low | 6 | 1.76(0.61-5.1) |  | 0.03(0-1.09) |  |
| Neural | High | 21 | 0.77(0.33-1.79) |  | 0.01(0-0.21) b |  | High | 22 | 0.7(0.3-1.63) |  | 0.01(0-0.38) a |  |
| Proneural | Low | 2 | 1.55(0.33-7.35) |  | 0.09(0-3.46) |  | Low | 2 | 1.54(0.33-7.32) |  | 0.06(0-2.03) |  |
| Proneural | High | 36 | 0.64(0.29-1.41) |  | 0.07(0-1.75) |  | High | 36 | 0.64(0.29-1.41) |  | 0.05(0-1.02) |  |
| Total | Low | 47 | 1.0 (ref.) |  | 1.0 (ref.) |  | Low | 35 | 1.0 (ref.) |  | 1.0 (ref.) |  |
| Total | High | 111 | 0.52(0.35-0.77) b |  | 0.49(0.33-0.74) c |  | High | 123 | 0.51(0.33-0.78) b |  | 0.5(0.32-0.77) b |  |

^a^*P* <0.05; ^b^*P* <0.01; ^c^*P* <0.001; ^1^The adjusted variables were the variables in adjust I.
