# Supplementary material for: Prognostic Value of mRNAsi/Corrected mRNAsi Calculated by the One-Class Logistic Regression Machine-Learning Algorithm in Glioblastoma Within Multiple Datasets
Source: Front Mol Biosci. 2021 Dec 6;8:777921. doi: 10.3389/fmolb.2021.777921 (PMC8685528; doi:10.3389/fmolb.2021.777921)
Supplement: Supplementary file 5 [file Table2.DOCX]

Table S2. Interaction test and stratified analysis in CGGA

|  | mRNAsi | | | | | | c_mRNAsi | | | | | |
| --- | --- | --- | --- | --- | --- | --- | --- | --- | --- | --- | --- | --- |
|  | Group | n | HR (95% CI) | *P* for intereraction | ^1^HR (95% CI) | *P* for interaction | Group | n | HR (95% CI) | *P* for interaction | ^1^HR (95% CI) | *P* for interaction |
| Age |  |  |  | 0.363 |  | 0.515 |  |  |  | 0.462 |  | 0.415 |
| <42y | Low | 16 | 1.0 (ref.) |  | 1.0 (ref.) |  | Low | 14 | 1.0 (ref.) |  | 1.0 (ref.) |  |
| <42y | High | 68 | 0.94(0.52-1.69) |  | 0.92(0.49-1.73) |  | High | 70 | 0.54(0.3-0.98) a |  | 0.48(0.25-0.93) a |  |
| 42y-54y | Low | 27 | 1.98(1.02-3.82) a |  | 1.48(0.58-3.76) |  | Low | 21 | 1.42(0.72-2.82) |  | 1.44(0.73-2.87) |  |
| 42y-54y | High | 74 | 1.12(0.63-2) |  | 0.89(0.36-2.22) |  | High | 80 | 0.71(0.4-1.26) |  | 0.76(0.42-1.39) |  |
| >54y | Low | 36 | 1.53(0.82-2.84) |  | 1.83(0.77-4.32) |  | Low | 26 | 0.86(0.45-1.66) |  | 0.94(0.49-1.82) |  |
| >54y | High | 58 | 0.93(0.51-1.69) |  | 1.52(0.61-3.78) |  | High | 68 | 0.65(0.36-1.17) |  | 0.73(0.4-1.32) |  |
| Total | Low | 79 | 1.0 (ref.) |  | 1.0 (ref.) |  | Low | 61 | 1.0 (ref.) |  | 1.0 (ref.) |  |
| Total | High | 200 | 0.66(0.5-0.88) b |  | 0.76(0.56-1.03) |  | High | 218 | 0.61(0.45-0.82) b |  | 0.61(0.45-0.84) b |  |
| Gender |  |  |  | 0.555 |  | 0.516 |  |  |  | 0.125 |  | 0.115 |
| Female | Low | 25 | 1.0 (ref.) |  | 1.0 (ref.) |  | Low | 21 | 1.0 (ref.) |  | 1.0 (ref.) |  |
| Female | High | 89 | 0.6(0.38-0.95) a |  | 0.67(0.41-1.1) |  | High | 93 | 0.45(0.27-0.73) b |  | 0.47(0.28-0.78) b |  |
| Male | Low | 54 | 0.9(0.56-1.47) |  | 0.99(0.5-1.98) |  | Low | 40 | 0.7(0.41-1.19) |  | 0.71(0.42-1.21) |  |
| Male | High | 111 | 0.64(0.41-1.01) |  | 0.81(0.4-1.64) |  | High | 125 | 0.51(0.32-0.81) b |  | 0.56(0.34-0.9) a |  |
| Total | Low | 79 | 1.0 (ref.) |  | 1.0 (ref.) |  | Low | 61 | 1.0 (ref.) |  | 1.0 (ref.) |  |
| Total | High | 200 | 0.67(0.51-0.89) b |  | 0.76(0.57-1.03) |  | High | 218 | 0.61(0.46-0.83) b |  | 0.66(0.49-0.9) b |  |
| IDH |  |  |  | 0.652 |  |  |  |  |  | 0.350 |  |  |
| No | Low | 77 | 1.0 (ref.) |  |  |  | Low | 58 | 1.0 (ref.) |  |  |  |
| No | High | 134 | 0.72(0.54-0.97) a |  |  |  | High | 153 | 0.62(0.45-0.85) b |  |  |  |
| Yes | Low | 2 | 1.15(0.28-4.68) |  |  |  | Low | 3 | 0.44(0.11-1.83) |  |  |  |
| Yes | High | 66 | 0.59(0.41-0.83) b |  |  |  | High | 65 | 0.52(0.35-0.75) c |  |  |  |
| Total | Low | 79 | 1.0 (ref.) |  |  |  | Low | 61 | 1.0 (ref.) |  |  |  |
| Total | High | 200 | 0.71(0.53-0.96) a |  |  |  | High | 218 | 0.65(0.48-0.88) b |  |  |  |
| Radiotherapy |  |  |  | 0.111 |  | 0.038 |  |  |  | 0.630 |  | 0.880 |
| No | Low | 17 | 1.0 (ref.) |  | 1.0 (ref.) |  | Low | 13 | 1.0 (ref.) |  | 1.0 (ref.) |  |
| No | High | 36 | 0.42(0.23-0.76) b |  | 0.36(0.18-0.73) b |  | High | 40 | 0.53(0.28-1) |  | 0.64(0.32-1.29) |  |
| Yes | Low | 62 | 0.4(0.23-0.69) b |  | 0.24(0.11-0.5) c |  | Low | 48 | 0.53(0.29-0.98) a |  | 0.53(0.29-0.99) a |  |
| Yes | High | 164 | 0.29(0.17-0.49) c |  | 0.2(0.09-0.41) c |  | High | 178 | 0.33(0.19-0.59) c |  | 0.36(0.2-0.65) c |  |
| Total | Low | 79 | 1.0 (ref.) |  | 1.0 (ref.) |  | Low | 61 | 1.0 (ref.) |  | 1.0 (ref.) |  |
| Total | High | 200 | 0.65(0.5-0.86) b |  | 0.71(0.53-0.96) a |  | High | 218 | 0.61(0.45-0.82) b |  | 0.67(0.5-0.91) a |  |
| Chemotherapy |  |  |  | 0.810 |  |  |  |  |  | 0.506 |  | 0.422 |
| No | Low | 17 | 1.0 (ref.) |  |  |  | Low | 11 | 1.0 (ref.) |  | 1.0 (ref.) |  |
| No | High | 32 | 0.73(0.4-1.32) |  |  |  | High | 38 | 0.75(0.38-1.47) |  | 0.87(0.42-1.78) |  |
| Yes | Low | 62 | 0.53(0.31-0.91) |  |  |  | Low | 50 | 0.6(0.31-1.15) |  | 0.6(0.31-1.16) |  |
| Yes | High | 168 | 0.36(0.21-0.59) c |  |  |  | High | 180 | 0.35(0.19-0.65) c |  | 0.38(0.2-0.71) b |  |
| Total | Low | 79 | 1.0 (ref.) |  |  |  | Low | 61 | 1.0 (ref.) |  | 1.0 (ref.) |  |
| Total | High | 200 | 0.68(0.52-0.9) b |  |  |  | High | 218 | 0.61(0.46-0.83) b |  | 0.67(0.49-0.91) b |  |
| MGMTP |  |  |  | 0.600 |  | 0.545 |  |  |  | 0.673 |  | 0.594 |
| No | Low | 42 | 1.0 (ref.) |  | 1.0 (ref.) |  | Low | 31 | 1.0 (ref.) |  | 1.0 (ref.) |  |
| No | High | 89 | 0.63(0.43-0.92) a |  | 0.69(0.46-1.04) |  | High | 100 | 0.66(0.43-1) |  | 0.72(0.47-1.11) |  |
| Yes | Low | 37 | 0.79(0.5-1.25) |  | 1.1(0.56-2.17) |  | Low | 30 | 0.97(0.58-1.62) |  | 0.97(0.58-1.63) |  |
| Yes | High | 111 | 0.57(0.39-0.84) b |  | 0.91(0.46-1.8) |  | High | 118 | 0.56(0.37-0.85) b |  | 0.6(0.38-0.92) a |  |
| Total | Low | 79 | 1.0 (ref.) |  | 1.0 (ref.) |  | Low | 61 | 1.0 (ref.) |  | 1.0 (ref.) |  |
| Total | High | 200 | 0.67(0.51-0.89) b |  | 0.75(0.56-1.01) |  | High | 218 | 0.62(0.46-0.83) b |  | 0.67(0.49-0.9) b |  |

^a^*P* <0.05; ^b^*P* <0.01; ^c^*P* <0.001; ^1^The adjusted variables were the variables in adjust I.
