# Supplementary material for: Prognostic Value of mRNAsi/Corrected mRNAsi Calculated by the One-Class Logistic Regression Machine-Learning Algorithm in Glioblastoma Within Multiple Datasets
Source: Front Mol Biosci. 2021 Dec 6;8:777921. doi: 10.3389/fmolb.2021.777921 (PMC8685528; doi:10.3389/fmolb.2021.777921)
Supplement: Supplementary file 7 [file Table5.DOCX]

Table S5. Brier score in CGGA

|  | Clinical | Clinical + mRNAsi | Clinical + c_ mRNAsi | mRNAsi | c_mRNAsi |
| --- | --- | --- | --- | --- | --- |
| C-index harrell |  |  |  |  | |
| apparent | 0.589 | 0.595 | 0.601 | 0.537 | 0.537 |
| optimism | 0.014 | 0.014 | 0.016 | 0.000 | 0.000 |
| adjust | 0.575 | 0.581 | 0.585 | 0.537 | 0.537 |
| C-index (0.5-year) |  |  |  |  | |
| apparent | 0.639 | 0.630 | 0.635 | 0.523 | 0.539 |
| optimism | 0.015 | 0.013 | 0.016 | 0.000 | 0.000 |
| adjust | 0.624 | 0.617 | 0.620 | 0.522 | 0.540 |
| C-index (1-year) |  |  |  |  | |
| apparent | 0.598 | 0.602 | 0.616 | 0.533 | 0.544 |
| optimism | 0.015 | 0.013 | 0.016 | 0.000 | 0.000 |
| adjust | 0.583 | 0.589 | 0.600 | 0.533 | 0.544 |
| C-index (1.5-year) |  |  |  |  | |
| apparent | 0.622 | 0.635 | 0.640 | 0.578 | 0.567 |
| optimism | 0.015 | 0.013 | 0.016 | 0.000 | 0.000 |
| adjust | 0.608 | 0.622 | 0.625 | 0.578 | 0.567 |
| C-index (3-year) |  |  |  |  | |
| apparent | 0.666 | 0.684 | 0.709 | 0.598 | 0.603 |
| optimism | 0.033 | 0.031 | 0.031 | 0.000 | 0.000 |
| adjust | 0.633 | 0.652 | 0.678 | 0.597 | 0.666 |
| C-index (5-year) |  |  |  |  | |
| apparent | 0.598 | 0.607 | 0.643 | 0.571 | 0.595 |
| optimism | 0.041 | 0.044 | 0.042 | 0.002 | 0.000 |
| adjust | 0.557 | 0.563 | 0.601 | 0.568 | 0.598 |
| Brier score (0.5-year) |  |  |  |  | |
| apparent | 0.143 | 0.143 | 0.143 | 0.154 | 0.153 |
| optimism | -0.004 | -0.004 | -0.004 | -0.001 | -0.002 |
| adjust | 0.147 | 0.147 | 0.147 | 0.155 | 0.155 |
| Brier score (1-year) |  |  |  |  | |
| apparent | 0.238 | 0.238 | 0.237 | 0.248 | 0.246 |
| optimism | -0.007 | -0.007 | -0.008 | -0.002 | -0.003 |
| adjust | 0.244 | 0.246 | 0.245 | 0.250 | 0.249 |
| Brier score (1.5-year) |  |  |  |  | |
| apparent | 0.220 | 0.218 | 0.217 | 0.225 | 0.225 |
| optimism | -0.007 | -0.007 | -0.008 | -0.003 | -0.002 |
| adjust | 0.227 | 0.224 | 0.225 | 0.228 | 0.228 |
| Brier score (3-year) |  |  |  |  | |
| apparent | 0.131 | 0.129 | 0.127 | 0.133 | 0.132 |
| optimism | -0.005 | -0.005 | -0.003 | -0.002 | -0.001 |
| adjust | 0.135 | 0.134 | 0.134 | 0.135 | 0.133 |
| Brier score (5-year) |  |  |  |  | |
| apparent | 0.094 | 0.094 | 0.093 | 0.095 | 0.094 |
| optimism | -0.004 | -0.004 | -0.002 | -0.001 | -0.001 |
| adjust | 0.098 | 0.098 | 0.096 | 0.096 | 0.095 |
