# Supplementary material for: Prognostic Value of mRNAsi/Corrected mRNAsi Calculated by the One-Class Logistic Regression Machine-Learning Algorithm in Glioblastoma Within Multiple Datasets
Source: Front Mol Biosci. 2021 Dec 6;8:777921. doi: 10.3389/fmolb.2021.777921 (PMC8685528; doi:10.3389/fmolb.2021.777921)
Supplement: Supplementary file 9 [file Table3.DOCX]

Table S3. Brier score in TCGA

|  | Clinical | Clinical + mRNAsi | Clinical + c_mRNAsi | mRNAsi | c_mRNAsi |
| --- | --- | --- | --- | --- | --- |
| C-index harrell |  |  |  |  |  |
| apparent | 0.664 | 0.685 | 0.683 | 0.576 | 0.563 |
| optimism | 0.015 | 0.019 | 0.017 | 0.000 | -0.001 |
| adjust | 0.649 | 0.666 | 0.667 | 0.576 | 0.564 |
| C-index (0.5-year) |  |  |  |  |  |
| apparent | 0.784 | 0.811 | 0.817 | 0.617 | 0.597 |
| optimism | 0.013 | 0.017 | 0.015 | -0.001 | -0.001 |
| adjust | 0.771 | 0.793 | 0.802 | 0.618 | 0.598 |
| C-index (1-year) |  |  |  |  |  |
| apparent | 0.695 | 0.745 | 0.733 | 0.618 | 0.584 |
| optimism | 0.013 | 0.017 | 0.015 | -0.001 | -0.001 |
| adjust | 0.682 | 0.727 | 0.718 | 0.618 | 0.585 |
| C-index (1.5-year) |  |  |  |  |  |
| apparent | 0.646 | 0.679 | 0.666 | 0.594 | 0.575 |
| optimism | 0.013 | 0.017 | 0.015 | -0.001 | -0.001 |
| adjust | 0.633 | 0.661 | 0.651 | 0.595 | 0.576 |
| Brier score (0.5-year) | |  |  |  |  |
| apparent | 0.145 | 0.139 | 0.142 | 0.180 | 0.181 |
| optimism | -0.008 | -0.009 | -0.007 | -0.003 | -0.004 |
| adjust | 0.154 | 0.148 | 0.148 | 0.183 | 0.185 |
| Brier score (1-year) |  |  |  |  |  |
| apparent | 0.204 | 0.195 | 0.199 | 0.227 | 0.232 |
| optimism | -0.010 | -0.012 | -0.012 | -0.006 | -0.005 |
| adjust | 0.214 | 0.207 | 0.210 | 0.233 | 0.238 |
| Brier score (1.5-year) | |  |  |  |  |
| apparent | 0.197 | 0.194 | 0.195 | 0.203 | 0.204 |
| optimism | -0.011 | -0.014 | -0.013 | -0.004 | -0.004 |
| adjust | 0.207 | 0.208 | 0.208 | 0.207 | 0.208 |
