# Supplementary material for: Prognostic Value of mRNAsi/Corrected mRNAsi Calculated by the One-Class Logistic Regression Machine-Learning Algorithm in Glioblastoma Within Multiple Datasets
Source: Front Mol Biosci. 2021 Dec 6;8:777921. doi: 10.3389/fmolb.2021.777921 (PMC8685528; doi:10.3389/fmolb.2021.777921)
Supplement: Supplementary file 10 [file Table4.DOCX]

Table S4. NRI and IDI in TCGA

|  | IDI | | | NRI | | |
| --- | --- | --- | --- | --- | --- | --- |
|  | 0.5-year (*P*) | 1-year (*P*) | 1.5-year (*P*) | 0.5-year (*P*) | 1-year (*P*) | 1.5-year (*P*) |
| Clinical (reference) | - | - | - | - | - | - |
| Clinical + mRNAsi | 0.03 (0.282) | 0.036 (0.126) | 0.021 (0.266) | 0.225 (0.09) | 0.222 (0.08) | 0.118 (0.264) |
| Clinical + c_mRNAsi | 0.019 (0.466) | 0.022 (0.282) | 0.014 (0.34) | 0.176 (0.2) | 0.156 (0.27) | 0.081 (0.36) |
| mRNAsi | -0.133 (0.018) | -0.096 (0.066) | -0.053 (0.268) | -0.319 (0.05) | -0.211 (0.22) | -0.141 (0.28) |
| c_mRNAsi | -0.137 (0.02) | -0.109 (0.042) | -0.061 (0.13) | -0.328 (0.056) | -0.188 (0.152) | -0.064 (0.384) |
