# Supplementary material for: Prognostic Value of mRNAsi/Corrected mRNAsi Calculated by the One-Class Logistic Regression Machine-Learning Algorithm in Glioblastoma Within Multiple Datasets
Source: Front Mol Biosci. 2021 Dec 6;8:777921. doi: 10.3389/fmolb.2021.777921 (PMC8685528; doi:10.3389/fmolb.2021.777921)
Supplement: Supplementary file 11 [file Table6.DOCX]

Table S6. NRI and IDI in CGGA

|  | IDI | | | | | NRI | | | | |
| --- | --- | --- | --- | --- | --- | --- | --- | --- | --- | --- |
|  | 0.5-year (*P*) | 1-year (*P*) | 1.5-year (*P*) | 3-year (*P*) | 5-year (*P*) | 0.5-year (*P*) | 1-year (*P*) | 1.5-year (*P*) | 3-year (*P*) | 5-year (*P*) |
| Clinical (reference) | - | - | - | - | - | - | - | - | - | - |
| Clinical + mRNAsi | 0.003 (0.693) | 0.002 (0.785) | 0.009 (0.222) | 0.01 (0.134) | 0.001 (0.793) | 0.008 (1.027) | 0.042 (0.643) | 0.121 (0.256) | 0.156 (0.11) | 0.025 (0.785) |
| Clinical + c_mRNAsi | 0.006 (0.531) | 0.007 (0.45) | 0.012 (0.196) | 0.016 (0.046) | 0.008 (0.312) | 0.053 (0.661) | 0.059 (0.462) | 0.101 (0.232) | 0.163 (0.048) | 0.061 (0.456) |
| mRNAsi | -0.053 (<0.001) | -0.05 (0.014) | -0.039 (0.076) | -0.028 (0.13) | -0.02 (0.308) | -0.202 (0.04) | -0.13 (0.088) | -0.111 (0.158) | -0.212 (0.324) | -0.016 (0.629) |
| c_mRNAsi | -0.049 (0.026) | -0.045 (0.064) | -0.038 (0.12) | -0.023 (0.184) | -0.023 (0.198) | -0.185 (0.09) | -0.111 (0.186) | -0.114 (0.126) | -0.126 (0.396) | -0.126 (0.356) |
